# Advancement in nanocarrier-mediated delivery of herbal bioactives: from bench to beside

**DOI:** 10.1007/s13659-025-00550-7

**Published:** 2025-11-01

**Authors:** Fatemeh Najafi, Negar Farrokhzad, Amirhossein Ghaemi, Dorsa Azizi Khezri, Mohammadali Hajiabbas, Amirhossein Khanizadeh, Nasim Kaveh Farsani, Mahsa Khoramipour, Niloofar Fatemipayam, Elham Seyyedi Zadeh, Arash Goodarzi, Behnoosh Khodadadi, Fatemeh Moradbeygi, Ahmad Reza Farmani, Mohammad Tavakkoli Yaraki, Martin Federico Desimone

**Affiliations:** 1 Department of Chemical Engineering, Pennsylvania State University, University Park, PA 16802-1503 USA; 2 Faculty of Biology, Medicine and Health, University of Manchester, Manchester, UK; 3 Department of Biotechnology, School of Chemical Engineering, College of Engineering, University of Tehran, Tehran, Iran; 4 School of Pharmacy, International Campus, Tehran University of Medical Sciences, Tehran, Iran; 5 Department of Chemical and Petroleum Engineering, Sharif University of Technology, Tehran, Iran; 6 Department of Chemical Engineering, Isfahan University of Technology, Isfahan, 84156-83111 Iran; 7 Department of Biology, Faculty of Sciences, Arak University, Arak, Iran; 8 Chemical Engineering Department, Oklahoma State University, Stillwater, OK USA; 9 Department of Tissue Engineering, School of Advanced Technologies in Medicine, Fasa University of Medical Sciences, Fasa, 74615-168 Iran; 10 Department of Chemistry, Faculty of Physics and Chemistry, Alzahra University, Tehran, Iran; 11 Department of Pharmaceutical Biotechnology, School of Pharmacy, Shiraz University of Medical Sciences, Shiraz, 71348-14336 Iran; 12 Pharmaceutical Sciences Research Center, Shiraz University of Medical Sciences, Shiraz, 71348-14336 Iran; 13 School of Natural Sciences, Faculty of Science and Engineering, Macquarie University, Sydney, NSW 2109 Australia; 14 Universidade Federal Do Rio Grande (FURG), Instituto de Ciências Biológicas (ICB), Programa de Pós-Graduação Em Ciências Fisiológicas (PPGCF), Rio Grande, RS Brazil; 15 Universidad de Buenos Aires (UBA), CONICET, Instituto de Química y Metabolismo del Fármaco (IQUIMEFA), Facultad de Farmacia y Bioquímica, Buenos Aires, Argentina

**Keywords:** Nano-delivery systems, Herbal compounds, Wound healing, Tissue engineering, Bioavailability

## Abstract

The resurgence of interest in traditional herbal remedies stems from an increasing appreciation for their complex phytochemical profiles and potential for synergistic therapeutic effects. However, the therapeutic potential of plant extracts is often limited by poor absorption and potential toxicity related to conventional delivery methods. This review explores the application of nanocarrier-mediated delivery systems, such as nanoparticles (NPs), liposomes, and nanoemulsions, to address these challenges. These biocompatible carriers offer enhanced stability and targeted delivery of herbal compounds, improving their efficacy and reducing unwanted side effects. By enabling precise distribution, nanotechnology optimizes the potency of herbal medicine across diverse applications, including regenerative medicine, wound healing, anticancer, and infection treatment. This review provides a systematic description of successful applications of nano-delivery technologies, nanoparticles, liposomes, nanoemulsions, and hybrid carriers, for the targeted delivery of some well-characterized herbal bioactives (curcumin, allicin, berberine, resveratrol etc.) and the enhanced therapeutic performance of herbal bioactives across a variety of preclinical models.

**Graphical Abstract:**

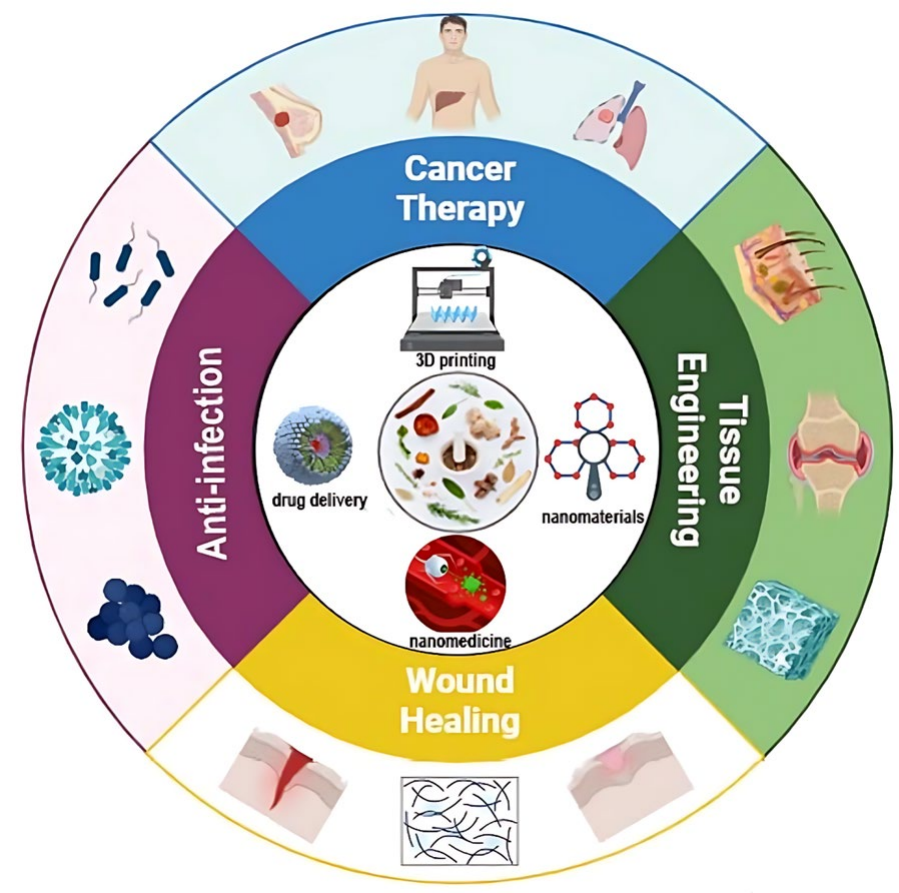

## Introduction

Medical remedies with botanical components have been used for more than five thousand years. Observations of animals consuming specific plants while sick most certainly inspired this behavior [1–4]. Although synthetic treatments accompany a wide spectrum of negative side effects, their creation helped mankind to evolve [5]. From somewhat minor issues like headaches or dizziness to more severe repercussions like cancer, cardiac arrest, or even death, these can range from rather minor worries [6]. Given this, traditional medicine has seen a comeback in its search for safer substitutes based on the great knowledge of herbal medicine gathered over years [7]. Many times seen as natural and risk-free options are herbal treatments [8]. Problems not immediately endangering a person's life are being treated with herbal therapy nowadays [9]. Among these ailments in men and women include cardiovascular disease, inflammation, depression, and problems of the reproductive system. Their potential usefulness, however, beyond the therapy and prevention of such diseases; herbal chemicals have showed promise as antibacterial agents as well as in the regeneration of tissue and the healing of wounds [10–12].

However, the therapeutic potential of plant extracts is often severely limited by significant pharmacokinetic challenges associated with conventional dosage forms like oral tablets and decoctions. These limitations are not merely theoretical but are quantifiably stark. For instance, regarding poor absorption and low bioavailability of curcumin, it should be noted that it is infamous for its extremely low oral bioavailability, often cited as less than 1% due to poor absorption, rapid metabolism, and systemic elimination [13, 14]. Similarly, the oral bioavailability of resveratrol is considerably less than 1% [15], and that of free genistein is 6.8% [16]. Decoctions, while traditional, suffer from inconsistent compound extraction, degradation of thermolabile compounds, and similarly poor gastrointestinal absorption.

Hence, the high doses required to overcome poor bioavailability often lead to unwanted side effects and organ toxicity. For example, high doses of free curcumin have been associated with hepatotoxicity and nephrotoxicity in preclinical models [17–19], while high concentrations of garlic-derived allicin can cause erythrocyte hemolysis and gastrointestinal irritation [20, 21]. Nanocarrier-mediated delivery systems directly address these quantified limitations, offering a demonstrable and superior alternative.

Moreover, nano-encapsulation drastically improves the absorption and bioavailability of herbal bioactives. For example, curcumin-loaded liposomes have shown a ninefold increase in oral bioavailability compared to curcumin suspended in water [22]. Piperine-loaded solid lipid nanoparticles (SLNs) demonstrated a 2.5-fold increase in bioavailability over a piperine solution [23]. A resveratrol-loaded nanoemulsion achieved an 3.2-fold higher relative bioavailability compared to a unformulated resveratrol suspension [24].

Additionally, by improving solubility and enabling targeted delivery, nano-systems allow for a lower effective dose, thereby reducing systemic toxicity. Berberine-loaded nanoparticles have been shown to significantly reduce cardiac and hepatic toxicity markers in animal models compared to free berberine at an equivalent dose [25, 26]. Furthermore, functionalized nanocarriers can facilitate active targeting, minimize off-site effects and enhance accumulation at the disease site (e.g., tumors, inflamed tissues) [27]. Furthermore, nano-encapsulation protects sensitive compounds from degradation in the harsh gastrointestinal environment [28] and enables controlled, sustained release, maintaining therapeutic drug levels for prolonged periods and improving patient compliance [29].

Concerns regarding the development of antibiotic resistance have focused attention on the direction toward natural drugs as replacements for manufactured antibiotics. Especially for disorders brought on by strains resistant to antibiotics, the research by Enioutina [30] emphasizes the effectiveness of molecules originating from plants in treating a spectrum of ailments. A lot of research has indicated that traditional treatments such garlic, honey, ginger, turmeric, and oregano have antibacterial properties.

In his research, Bussmann [31] have discovered that several plant species used in folk medicine for disease management actually exhibit antibacterial properties. Albaridi [32] highlights the usefulness of honey in combating bacteria. It has been stressed by Tayel [33] and Combarros-Fuertes [34] that culinary botanicals like oregano demonstrate efficacy against antibiotic-resistant bacteria and foodborne pathogens. Chemicals that are generated from medicinal herbs and nutritious plants have shown a great deal of promise in the management and prevention of cancer. The reason for this can be ascribed to the several pharmacological properties that they possess, which include anti-inflammatory, anti-proliferative, and antioxidant properties [35, 36]. Strong anticancer properties have been shown for many herbal constituents including turmeric, resveratrol, genistein, and others. these elements stop the spread of cancer cells, cause programmed cell death, and slow down tumor growth [37–39].

**Fig. 1:**
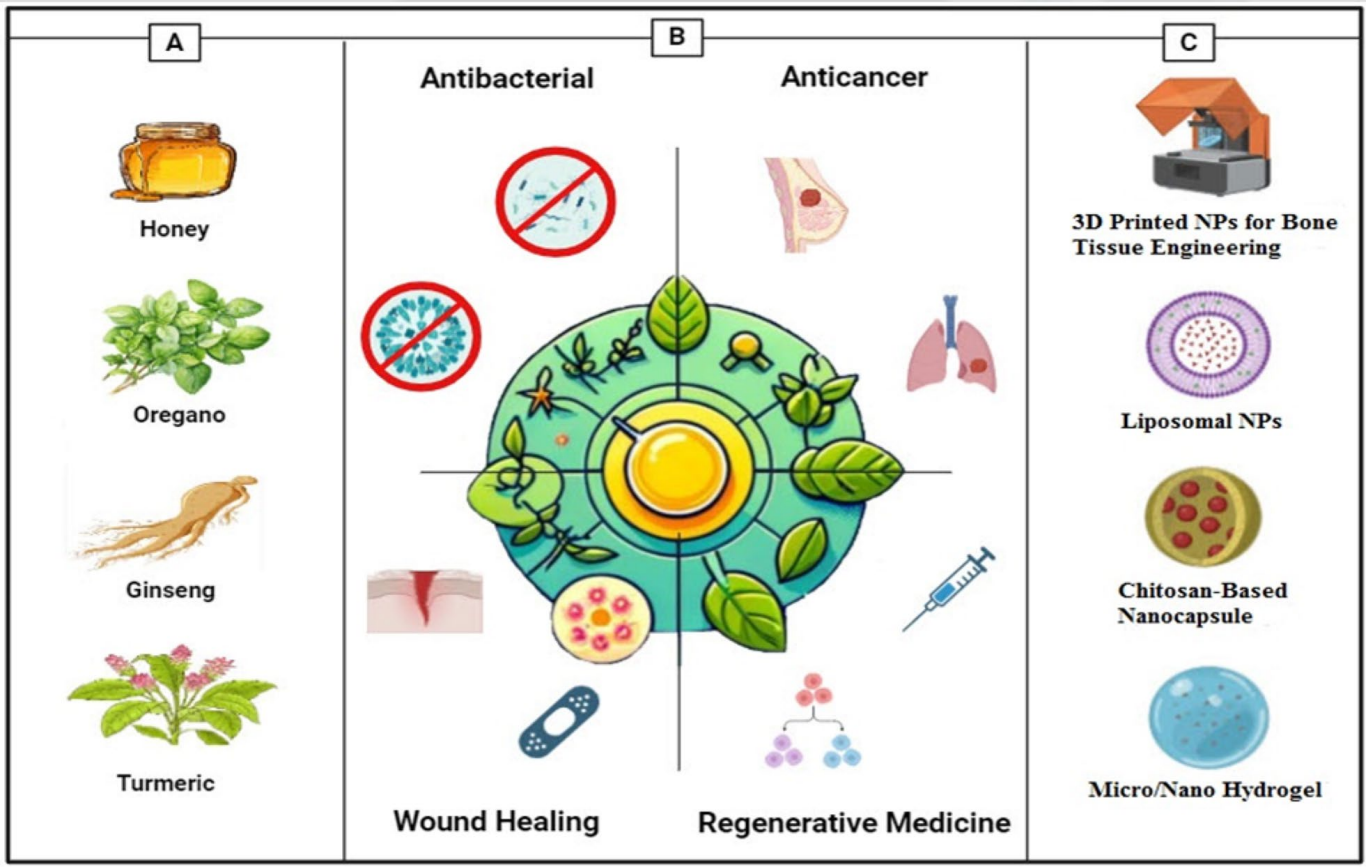
Overall view of this report. **A** The commonly used plants and natural compounds and their bioactive compound [Honey–Turmeric–Ginseng–Oregano] **B** Main applications [Antibacterial–anticancer–wound healing–regenerative medicine] **C** Main delivery systems

When it comes to medical therapy, wound healing is of the utmost importance, not only for the purpose of physical recovery but also for the purpose of psychological well-being [40, 41]. It is essential to provide appropriate wound care in order to avoid problems such as infections, deformed scars, and prolonged hospitalization from occurring [42]. Phenolic compounds, alkaloids, saponins, and flavonoids among the drugs demonstrated to boost collagen generation also cell proliferation and angiogenesis [43]. Especially remarkable is the evidence showing *Allium sativum*, Aloe vera (AV), *Centella asiatica*, and *Hippophae rhamnoides* greatly increases burn injury repair [44]. It has been demonstrated that herbal medicines that are used topically have the potential to be effective in treating burn wounds; however, additional research is required to evaluate the possibility of harmful consequences [45]. Moreover, herbal compounds have demonstrated potential in the field of tissue engineering (TE) and regeneration. Li's study found that polysaccharides produced from Chinese medicinal herbs help wounds heal and tissue damage be repaired [46]. It is commonly accepted that Chinese herbal medicines have the potential to promote adult stem cells in the process of tissue regeneration [47]. There is considerable research being undertaken on the potential applications of plant-derived biomaterials for tissue engineering [48]. Apart from motivating bone regeneration, phytochemical compounds including flavonoids, tannins, and polyphenols can be integrated into nanostructured scaffolds with the purpose of bone tissue engineering [49]. On the other hand, poor digestion and absorption lowers the efficacy of herbal remedies by insufficient active components reaching their intended purpose. Moreover, at appropriate dosages, herbs could be allergenic or poisonous, thereby impairing organs or causing nausea or vomiting [50, 51]. Bettering delivery methods will help to make herbal medicines safe and efficient for life-threatening diseases. Using nanodelivery technologies more focused and less side effects treatment solutions are accessible [52]. NPs based on proteins, lipids, or polysaccharides can show antimicrobial effect; regardless of their basis [53]. This is accomplished by creating osmotic stress in microbial cells, which increase membrane permeability [51]. Including NPs into various carriers, including hydrogels, increases their adhesion to the surfaces of tissues, so enabling highly concentrated treatment to be more realistic [54, 55]. While albumin-based nanocarriers enhance drug absorption in the body due to their widespread presence in humans and excellent biocompatibility, cellulose-based NPs improve the morphology of delivery vesicles and enhance their high cytocompatibility [56, 57]. It is possible to preserve herbal compounds against degradation using nano-delivery carriers such as nanoemulsions and liposomes, which also ensure great bioavailability and biocompatibility [58]. Figure 1 offers a schematic representation of the report's content. Panel A illustrates commonly used plants and natural compounds with bioactive properties. Panel B outlines the main applications, spanning antibacterial, anticancer, wound healing, and regenerative medicine. Panel C highlights the main delivery systems employed in herbal medicine research.

The aim of this review is to thoroughly examine the characteristics of active substances derived from plants and their prospective uses in treating human ailments, focusing specifically on their antibacterial effect, anticancer effect, wound healing properties, and their role in tissue engineering. This work further explores the application of nano-carriers, including nanoparticles, liposomes, and nanoemulsions, to enhance the delivery efficiency of herbal medicines. The emergence of groundbreaking developments in herbal medicine has facilitated the creation of safer and more efficacious therapies for numerous ailments. Through a rigorous analysis of these advancements, this review seeks to elucidate their transformative potential for medical care.

## Antibacterial applications

The emergence of antibiotic-resistant bacteria has motivated study on innovative approaches to fight bacterial diseases. Including herbal components into nanodrug delivery systems helps fight antibacterial agents holistically and ecologically friendly [59]. By increasing drug absorption and lowering therapy and side effect resistance, stimulus-responsive drug delivery and therapeutic nanoparticles can help to improve these systems [60]. In these systems, nanocarriers can improve the stability, precise transportation, and bioavailability of herbal substances [61]. Furthermore, studies have shown that, particularly in terms of targeted delivery, environmentally sensitive transportation, and combinatorial administration, nanoparticle drug delivery methods can increase the therapeutic efficacy of present antibiotics [62].

Hence, in the current section, we selected herbal compounds based on certain scientific and translational criteria. We selected evidence-based natural products like allicin, honey products, gingerol, curcumin, and carvacrol, generally recognized for effectiveness against a variety of microbial species supported by pharmacological studies [63, 64]. These bioactives have been incorporated in different types of nano-delivery systems and researched both in vitro and in vivo, resulting in a good representation of compounds for demonstrations of enhancing antimicrobial effects through nanocarrier-mediated techniques [65]. The selections also provide some structural diversity within specific phytochemical classes, which allows for discussion on how the delivery systems may differentiate chemical properties.

This section explores the potential of herbal compounds wide range of phytochemical classes (i.e., polyphenol, flavonoid, alkaloid, organosulfur compounds, terpenoids) as antibacterial agents within nanodrug delivery systems to revolutionize infection treatment.

### Antimicrobial herbal compounds

#### Garlic

Garlic, formally known as *Allium sativum Linn*, has been valued for millennia for its remarkable medicinal properties including great antibacterial action [51]. Key in garlic's antibacterial action is allicin (AC), a bioactive molecule first discovered in 1944 by Cavallito and Bailey [66].

Against both gram-negative and gram-positive bacteria, garlic-derived AC shows a broad-ranging antibiotic arsenal [67]. Moreover, AC has showed antiviral and antifungal action against *Candida albicans* as well as antiparasitic properties against human intestinal protozoan parasites like *Giardia lamblia* and *Entamoeba histolytica* [67]. The importance of allicin in the antibacterial properties of garlic cannot be overstated. Indeed, garlic's antibacterial effects are lowered in the absence of AC or when alliinase, the enzyme in charge of allicin synthesis, is suppressed [68, 69]. Allicin's inhibitory effects on bacterial growth are comparable, if not better than several conventional antibiotic such as penicillin, tetracycline [70], and kanamycin [71, 72]. Especially, allicin's inhibitory properties cover a broad spectrum of microorganisms including bacteria, yeasts, fungus, and parasites [73, 74]. AC's multifarious and strong antimicrobial action makes it a desirable choice for nano-delivery systems to improve antimicrobial efficacy. Hasemy and coworkers used chitosan (CS)-lecithin nanoparticles modified with polyethylene glycol (PEG) and folic acid (FA) to deliver AC to colon cancer cells. Their study revealed that treatment with these nanoparticles reduced the viability of colon cancer cells. Also, in this study the human foreskin fibroblast was used as normal cells [75]. Allicin makes quite flexible use of its antimicrobial qualities. It can therefore affect the vital bacterial activities, where it reduces previously present walls and hinders cell wall formation. Moreover, it disturbs metabolism in bacteria and inhibits ribosome and enzyme activities, therefore upsetting protein synthesis and DNA replication. Especially, allicin's mode of action has led to the hypothesis that establishing resistance against it may be notably 1000-fold more difficult compared to beta-lactam antibiotics [76]. Studies have found that AC mostly targets RNA synthesis and somewhat reduces DNA and protein synthesis. Reactive oxygen species (ROS) produced by this complex interference with microbial operations finally cause damage to DNA and proteins and culminate in microbial cell death [51].

#### Honey

The bees feed on flower nectar or blossoms that can later on is made into honey, which contains water, and a high number of sugars, such as glucose or fructose (~ 80% of its weight), however, it also has present compounds that can help treat bacterial infection. It contains vitamins, amino acids, enzymes, and a few vital minerals, but the main antibacterial component of the honey is hydrogen peroxide (H_2_O_2_) and bee defensin-1. The composition of the honey’s active compounds varies, depending on the plant and its part it has been collected from. For example, pure honey will have different antimicrobial properties, compared to multifloral honey [77]. Additionally, its high osmolarity and acidic pH create a mixture that has been used to treat bacterial infections [78]. H_2_O_2_ is produced through the oxidation of glucose, which is catalysed with glucose oxidase from bees, which is damaging to the bacterial cells by oxidation of the membrane and genetic material, and together with acidic pH, it prevents bacterial growth. This substance is widely used for disinfection purposes, not only at home but also in high concentrations (0.8-8M) can be used in medical settings (Stan et al., 2021). H_2_O_2_ has been proven to work against a few of the most common bacterial genera and their species, such as S*taphylococcus* spp., *Streptococcus* spp., *Bacillus* spp., and more medically significant microbes. Furthermore, some types of honey contain methylglyoxal (MGO), which reduces the motility of some bacteria, such as *Pseudomonas aeruginosa,* through deflagellation, however it can also disrupt fimbriae in other bacteria species [79]. MGO was reported to successfully retard the expansion of various bacteria, both, gram-positive and negative, by reducing its adhering capabilities through alternation of their structure [80, 81]. Moreover, in another study, researchers assessed the antibacterial effects of Black Forest honeydew and manuka honey UMF 20 + against *Escherichia coli* and *Staphylococcus epidermidis*, identifying inhibitory activity at 10–30 v/v% concentrations and higher gluconic acid content in manuka honey. Manuka honey was incorporated into polycaprolactone nanofiber scaffolds via pressurized gyration, producing fibres averaging 437–815 nm. These scaffolds exhibited over 90% bacterial reduction against *S. epidermidis*, demonstrating strong antibacterial efficacy. The findings suggest that honey-based composite nanofibers have significant potential as natural therapeutic agents, particularly in wound healing [82].

#### Ginger

**Fig. 2:**
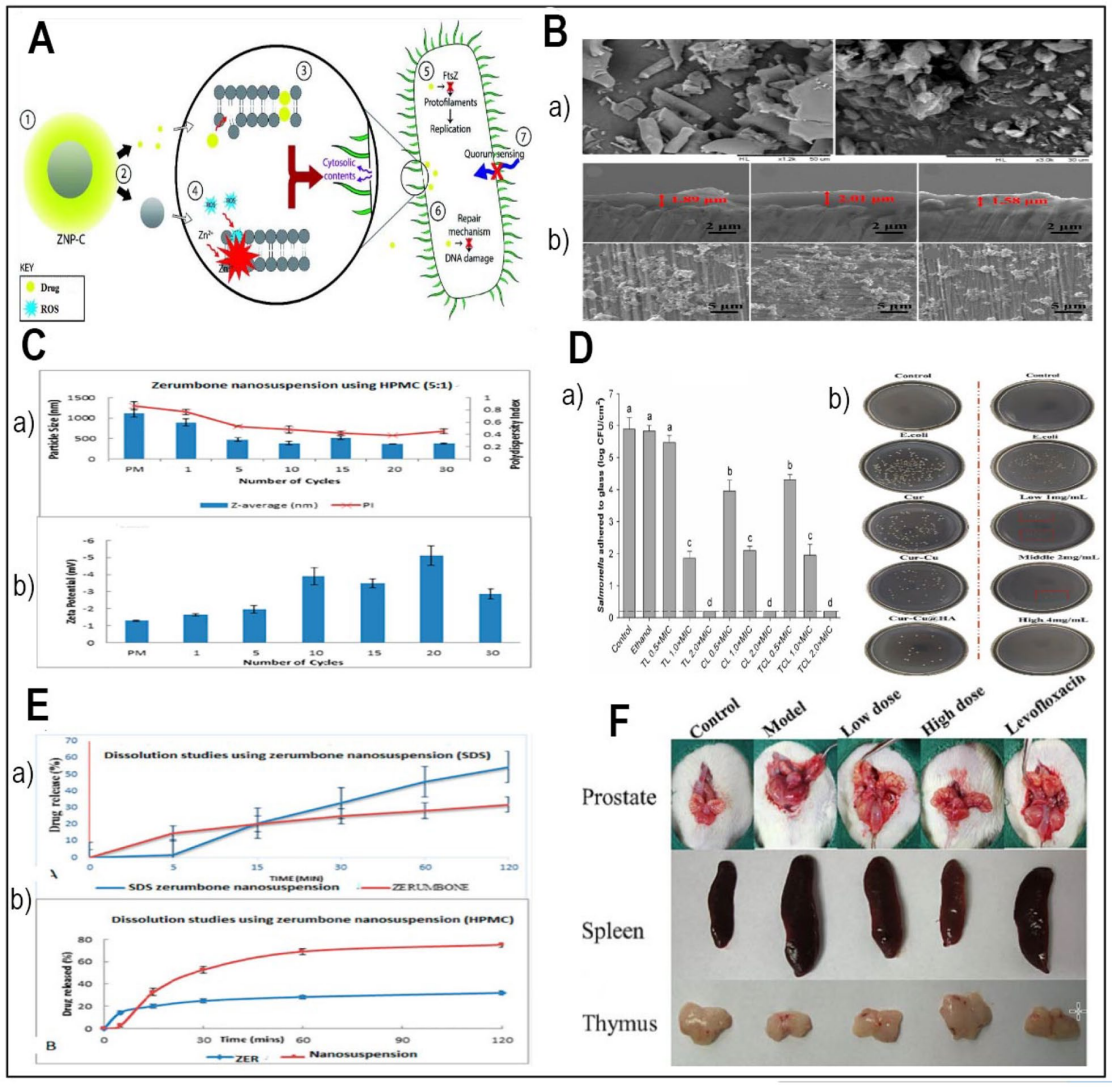
**A** The synergistic impact of the nanocarrier-drug combination on bacteria (Redesigned from reference [89] with permission). **B** SEM images of freeze-dried nanosuspensions containing SDS-zerumbone (Left) HPMC-zerumbone (Right) (Redesigned from reference [86] with permission). **C** The particle size and size distribution (measured as polydispersity index (PI) (**a**) and the zeta potential (**b**) of zerumbone nanosuspensions stabilized by HPMC decrease with increasing homogenization cycles (Redesigned from reference [86] with permission). **D** Effects of encapsulated antimicrobials, after a 1-min contact with Salmonella adhered to glass surface (**a**) (Redesigned from reference [95] with permission), The plate colony images of ALL@CS incubating with E. coli for about 4 h (**b**) (Redesigned from reference [101] with permission). **E** The dissolution profiles of (**a**) a freeze-dried nanosuspension stabilized by SDS and (**b**) a freeze-dried nanosuspension stabilized by HPMC were evaluated in a phosphate buffer solution at pH 7.4 and a temperature of 37 °C [86]. **F** The therapeutic efficacy of Cur-Cu@HA for prostatitis in rats. Images of the prostate, spleen, and thymus in various categories.(Redesigned from reference [101] with permission). (p-value = p, *p < 0.05, **p < 0.01, *** p < 0.001)

Renowned for its many culinary and medicinal uses, ginger has a strong toolkit of antibacterial properties awaiting for nano-delivery systems (NDS). The bioactive chemical gingerol, which has great potential to fight infections especially in the oral cavity, is central in ginger's antibacterial effectiveness. The antibacterial mechanism of gingerol goes beyond accepted wisdom. Crucially important bacterial activities including adhesion, biofilm development, and quorum sensing—all of which contribute significantly to bacterial virulence—are stopped here. By upsetting these systems, gingerol shows promise as a creative antibacterial agent [83]. Furthermore, zerumbone, a less well-known component of ginger originally taken from wild ginger, shows a broad spectrum of therapeutic effects including anti-inflammatory, antibacterial, antiplatelet, antifungal, cytotoxic, and chemopreventive qualities [84]. However, zerumbone has always struggled with low water solubility, poor absorption, limited bioavailability, and difficulties getting the molecule to target tissues. The relevance of zerumbone has changed with the invention of nano-delivery techniques. Several investigations including those by Foong et al. and Md et al. have showed notable increases in the oral bioavailability and medicinal efficacy of zerumbone by means of NDS [85, 86] (Table 1). Md and colleagues developed a nanosuspension form of zerumbone in 2018 in order to boost solubility of it. In high-pressure homogenizing process, stabilizers were sodium dodecyl sulfate (SDS) and hydroxypropylmethylcellulose (HPMC).. Aggregation was absent in the zerumbone nanosuspensions stabilized by SDS presumably because of their strong zeta potential. Still, while looking at zerumbone nanosuspensions stabilized by HPMC, they discovered aggregated crystals most likely from low zeta potential. Scanning electron microscopy (SEM) images revealed particle agglomeration in both nanosuspension formulations resulting from water removal [86]. Emphasized in Fig. 2C, notable variations in the mean particle size and zeta potential of zerumbone nanosuspensions stabilized by SDS and HPMC showed themselves. Specifically, the mean particle size of the nanosuspension stabilized by SDS was 211 ± 27 nm and the zeta potential was about − 30.9 mV, whereas those stabilized by HPMC showed a mean particle size of almost 400 nm and a zeta potential of −3.37 mV. As shown in Fig. 2E, the dissolving properties of the untreated zerumbone were evaluated against those of the zerumbone nanosuspensions produced with SDS or HPMC stabilizers. Using SDS, the drug release from nanosuspensions was much higher than that of its equivalent (54.6% ± 7.3 vs. 31.9% ± 1.2). Comparatively to the crude zerumbone (75% ± 2.2 vs. 31.9% ± 1.2), the nanosuspensions of zerumbone, generated using HPMC, showed a clearly higher drug release percentage. The results show that nanosizing has enhanced zerumbone's solubility properties [86]. These difficulties of solubility, absorption, and targeted distribution have been essentially overcome by encapsulating zerumbone into nano-carriers, therefore opening new options for the use of ginger's antibacterial treasure store in the fight against microbial diseases.

**Table 1:** Summary of studies in the existing literature utilizing nano-delivery systems for herbal compounds for antibacterial application

Name of the compound	Bioactive compound	Nanodelivery method	Fabrication of the carrier	In vitro and in vivo assays	Cell lines and strains used	Refs.
Garlic	Allicin	Chitosan-lecithin nanoparticles modified with PEG and FA	Self-assemblingprocedure	Kirby-Bauer Disk Diffusion, MIC method, etc	*Two gram-positive (S. aureus* and *B. subtilis)* and two gram-negative (E. *coli* and *P. aeruginosa)*	[75]
	Allicin	Polydopamine/tannic acid-allicin@chitosan	Microemulsionmethod	The colony counting method	*E. coli* or *S. aureus*, *P. aeruginosa*	[99]
Honey	Honey bee propolis extract	Carrageenan (κ-CAR) and β-cyclodextrin (β-CD)		Zone of inhibition assays	Gram-positive (*S. aureus)*, gram-negative (*P. aeruginosa*), *fungus* (*Candida albicans*), and yeast (*Aspergillus niger*)	[55]
Ginger	Zerumbone	Nanosuspension	High-pressure homogenization		Antibacterial activity on *S. choleraesuis*	[86]
Turmeric	Curcumin	Curcumin-propylene glycol liposomes		Identification and quantification of the bacteria were started with taking swabs		[100]
	Curcumin	ZnONPs and ZnONCs	Aqueous solutions of zinc nitrate hexahydrate	Zone of inhibition assay—disc- and well diffusion agar methods	Gram-positive (*S. aureus* (ATCC 25923), S. epidermidis (clinical isolate), B. cereus (ATCC 9027)) and one Gram-negative bacterium (*E. coli* (ATCC 35218))	[89]
	Curcumin	Curcumin copper complex grafted with hyaluronic acid	Esterification reaction between the hyaluronic acid and cur-cu	Plate counting method	*E. coli*	[101]
Oregano	Thymol			Zone of inhibition assays and MIC method	Gram-positive (Bacilluscereus, Listeria monocytogenes, *S. aureus* and *E. faecalis*) and Gram-negative (Salmonella entericaserovar Typhi*, E. coli*, Shigella flexneri, P. aeruginosa, Proteus mirabilis and Klebsiella pneumoniae)	[94]
	Carvacrol	BSA on a chitosan core		Simulated oral, gastric, and intestinal digestion	*Salmonella enterica*	[97]
	Carvacrol	Pectin-alginate microcapsules	Spray drying method	Evaluation of the minimum inhibitory concentration (MIC)	*E. coli*	[102]
	Carvacrol	Carvacrol-loaded nanoemulsions	Box-Behnken design	Scanning electron microscopy, reactive oxygen species, etc	*S. aureus and E. coli*	[103]
	Thymol and Carvacrol	Co-delivery of thymol and carvacrol with nanoliposome		Microbroth Dilution—MIC	S. enterica	[95]
	Carvacrol	Co-delivery of carvacrol and astaxanthin with beeswax SLNs	Response surface methodology	Microbroth Dilution—Evaluation of the minimum inhibitory concentration (MIC)	*Pseudomonas aeruginosa S. aureus*	[104]
	Carvacrol	Carvacrol loaded with propylene glycol monopalmitate and glyceryl monostearate	Microemulsion template method	Microbroth Dilution—Evaluation of the MIC	*S. aureus* and *E. coli*	[105]
	Carvacrol	Chitosan-cv nanoparticles		Bacteria Viability	*S. aureus* and* E. coli*	[106]
	Carvacrol	Rhamnolipid-stabilized CV-loaded zein		Broth dilution—Evaluation of the MIC	*Fusarium oxysporum*	[107]
		CV‐loaded zein nanoparticles		Broth dilution—Evaluation of the MIC	*S. typhimuriumP. aeruginosaS. aureus*	[108]
	Thymol	TM-loaded zein/shellac	Coaxial electrospray process	Disk diffusion	*S. aureus* and *E. coli*	[109]
		Polyethylenimine (PEI)-coated CV	Nanoprecipitation methods	Microbroth Dilution—Evaluation of the minimum inhibitory concentration (MIC)	L. monocytogenes, E. coli, P. aeruginosa, S. enterica, S. aureus	[110]

#### Turmeric

Renowned for both its vivid colour and many medical benefits, turmeric contains curcumin (Cur), a naturally occurring polyphenol antioxidant with a β-diketone [87]. With great ramifications for antibacterial treatment, curcumin's several antibacterial processes have attracted much study attention. Antimicrobial repertory of curcumin spans several groups of pathogens, including bacteria, viruses, fungus, and parasites [88]. Its potency results from its capacity to alter gene expression, affect energy metabolism, and damage bacterial membranes. Though it has great potential, curcumin struggles with low stability, high metabolism, poor water solubility, and quick elimination. Researchers have developed several nano-delivery systems aiming at curcumin's solubility, targeting, and antibacterial action to overcome these challenges. While zinc oxide nanoparticles can increase its antibacterial activity [89], liposome nanocarriers can improve curcumin's transdermal distribution and sustained release [88] (Table 1). Recent research [89] has revealed a new delivery method, Cur-Cu@Hyaluronic acid (HA), which mixes curcumin with copper ions and hyaluronic acid, efficiently treats bacterial prostatitis, a painful and incapacitating illness brought on by an *E. coli* infection. These findings highlight the significant possibilities of curcumin-based nano-delivery technologies for the fight against bacterial diseases.

#### Oregano

Popular for its aromatic essential oils, oregano has strong antiseptic and antibacterial qualities [90]. Among its array of bioactive chemicals, two very well-known ingredients—carvacrol and thymol—have attracted a lot of interest for their antibacterial properties and several modes of action. A main component of oregano oil, carvacrol targets bacterial cell walls and membranes to mostly show antibiotic activities [91, 92]. It compromises the integrity of the outer and inner membranes, therefore lowering the oxidative stress tolerance in bacteria and compromising enzyme activity. Furthermore, carvacrol positions itself within lipid chains by modifying the flexibility and penetrability of the cell membrane, therefore proving its efficiency. This impairment of cell homeostasis causes a drop in membrane potential and intracellular pH. Moreover, phytochemical substances including carveol, carvone, citronellol, and citronellal can elicit cell lysis by delocalizing electrons and generating the continuous release of K^+^ ions outside the membrane, therefore resulting in bacterial cell death [93].

Another well-known component of oregano, thymol shows antibacterial action by encapsulating in different nano-sized delivery vehicles [94] (Table 1). In a study undertaken by Caroline Heckler and colleagues in 2020, the antimicrobial activities of nanoliposome-encapsulated thymol, carvacrol, and a combination of thymol/carvacrol (1:1) were evaluated against a pool of *Salmonella bacteria*. Promising features of the encapsulated antimicrobial agents were excellent encapsulation efficiencies (~ 99%) and nanoliposomes with sizes between 230 and 270 nm. For both free and encapsulated antibiotics, Fig. 2D part a shows the notable declines in adherent *Salmonella* populations at the minimum inhibitory concentration (MIC). While ethanol indicates the surviving *Salmonella bacteria* following one minute of contact to a 20% ethanol solution, control is the population of *Salmonella bacteria* adhered to the glass surface. Every bar in the figures shows the three separate, unconnected standard deviation of three different experiments. Different letters stand for statistically significant variations (p < 0.05). The dotted line shows 0.2 log of colony-forming units per square centimeter, the detection limit of this technique. As shown in Fig. 2D part a, encapsulated antibiotics totally eradicated adhering *Salmonella* at a concentration 2.0 times the MIC. With no clear variation across the liposomal preparations, MIC showed considerable declines (3.79–4.03 log CFU/cm^2^; p < 0.05) [95].

Research have effectively captured important phytochemicals, including thymol, in liposomes showing synergistic antibacterial action against several bacteria [96]. Characteristic of effective trapping of active chemicals, the liposomal systems had tiny diameters of roughly 144 nm and negative zeta potentials of roughly − 30 mV. Moreover, great encapsulation efficiency and sustained release characteristics have been described by means of the encapsulation of thymol inside cyclodextrins and its derivatives [93]. Concurrent with this development of corona-modified NDS using bovine serum albumin (BSA) on a CS core, carvacrol has found its niche in nano-delivery systems (Table 1) [97]. These NDS provide a forum for the continuous release of the respected natural antibacterial agent carvacrol into the intestine. With exact control and targeted distribution, this creative strategy provides means to maximize the antibacterial power of carvacrol. A notable study investigated the antibacterial efficacy of previously synthesized and characterized chitosan nanoparticles loaded with carvacrol, assessing their synergistic effects with Topoisomerase II inhibitors—ciprofloxacin and doxorubicin—against *Staphylococcus aureus*, *Escherichia coli*, and *Salmonella typhi*. The findings demonstrated that combining carvacrol nanoparticles with these antibiotics significantly lowered the minimum inhibitory concentration (MIC) compared to the drugs alone. Specifically, ciprofloxacin exhibited MIC_50_ values of 35.8 µg/mL, 48.74 µg/mL, and 35.57 µg/mL, while doxorubicin showed MIC_50_ values of 20.79 µg/mL, 34.35 µg/mL, and 25.32 µg/mL against *S. aureus*, *E. coli*, and *S. typhi*, respectively, highlighting enhanced antibacterial activity through nanoparticle-mediated synergy. This study revealed that herbal loaded nanoparticles can also, help as a subsidiary treatment for traditional antibiotics [98].

### Delivery of antibacterial herbal compounds

Because they could be efficient substitutes or complements for conventional antibiotic treatment, antibacterial plant substances have attracted major interest in recent years. Conventional approaches of delivering herbal medications, such oral supplements, have restrictions in terms of attaining localized and effective distribution to target sites [80]. As suggested by Stan et al., a potential method to get above these constraints is the exact delivery of bioactive substances made possible by nanocarriers. The several nanoparticle formulations utilized for the transport of antibacterial herbal components are covered in this part together with their ingredients and possible uses. Figure 2A shows how the nanocarrier-drug combination acts in concert to kill bacteria.

#### Nanoparticulate systems for antibacterial drug delivery

Nanoparticulate systems offer a versatile framework for the delivery of herbal medications having antibacterial action. Various materials can be used in engineering these nanocarriers to satisfy particular therapeutic goals. Key characteristics of nanoparticles, including their form, size, and surface charge, can be precisely modified by changing material types, contents, and manufacturing procedures, thus optimizing drug release kinetics and obtaining exact organ or cell targeting [111]. For example, garlic's phytoconstituents, among others, have shown promise in antimicrobial uses; issues with solubility, stability, and bioavailability have hampered their practical relevance. By means of nanocarriers like NPs, liposomes, carbon nanotubes, quantum dots, and hydrogels, nanotechnology has become a way to solve these problems (Prajapati et al., 2021). These nanocarriers not only increase solubility and stability but also target capacity of phytoconstituents, hence enhancing therapeutic results and lowering the needed dosage. Typically researched for antibiotic treatments are inorganic metal nanoparticles, polymeric nanoparticles, lipid-based nanoparticles, micellar nanoparticles, silica nanoparticles, and cell membrane-coated nanoparticles [112, 113].

##### Chitosan

Considered a good source of pH-responsive nanocapsules, cationic natural polysaccharide chitosan has intrinsic antibacterial effect. Protonation and deprotonation of the amino groups generates this in chitosan. Hao et al., (2022) investigated the prospect of chitosan-based nanocapsules as carriers for antibacterial herbal components, therefore offering a twofold advantage of natural antibacterial action and regulated drug release. Figure 2D part b shows the effectiveness of the antibacterial properties against *E. coli* varies depending on the pH settings. The data demonstrate that the colonies exhibit a greater abundance when exposed to solutions with pH levels of 8 and 7, as opposed to solutions with pH levels of 6 and 5. Furthermore, the number of colonies is at its minimum when the solid medium is incubated in pH 5 phosphate-buffered saline (PBS) settings. The results indicate that the acid-allicin@chitosan (ALL@CS) nanocapsules demonstrate enhanced antibacterial efficacy in acidic conditions [99] (Table 1).

##### Hyaluronic acid

Because of its main sodium salt form, hyaluronic acid (HA), a big glycosaminoglycan molecule found abundantly in the human body, offers excellent biocompatibility and high water-solubility [114]. As the CD44 receptor, which shows large expression levels in inflammatory areas and participates in inflammation, hyaluronic acid is the ideal ligand for focused medication distribution [115, 116]. Hyaluronic acid-based nanocarriers have showed great promise in delivering antibacterial herbal components to particular locations of illness according to many research [117, 118]. This method minimizes off-target effects, hence improving drug accumulation at the intended site [101] (Table 1). In 2023, Gao and colleagues developed a targeted drug delivery system by grafting a curcumin-copper complex onto hyaluronic acid (HA). Targeting ability, antibacterial activity, and carrier solubility were among the aspects where the planned delivery system most certainly enhanced. Grafting hyaluronic acid enhanced water solubility and gave the carrier amazing CD44 receptor targeting ability. Additionally, the complexation of copper ions with the carrier significantly improved its antibacterial properties, particularly its inhibitory effect on *E. coli*. (Fig. 2D part B). In vivo study of the anti-prostatitis effects showed that by lowering inflammation, the Cur-Cu@HA delivery method effectively promoted recovery. Figure 2F shows the variations in the prostate circumstances among the experimental groups. The prostate in the experimental cohort was clearly enlarged and had a denser texture than in the normal group, suggesting effective modeling. Prostate of the two groups treated with Cur-Cu@HA, particularly at high dosages, Cur-Cu@HA shown better therapeutic efficacy than levofloxacin [101]. The Cur-Cu@HA administration method showed good possibilities for treating bacterial prostatitis. Combining focused administration, improved solubility, and strong antibacterial action, its multifarious approach points to it as a possible treatment choice for this illness [101].

#### Impact of physicochemical properties of nanocarriers on antibacterial efficacy

The antibacterial efficacy of nano-delivery systems depends not only on the encapsulated bioactive compound but is also significantly affected by the nanocarrier's physicochemical properties, such as particle size, surface charge (zeta potential), and polydispersity index (PDI) [119, 120]. These factors influence nanoparticle stability, bacterial interaction, biofilm penetration, and release behavior. Smaller nanoparticles, typically under 100 nm, demonstrate improved cellular uptake and deeper biofilm penetration, which aids in combating persistent infections [121]. For example, a zerumbone nanosuspension stabilized by SDS with an average size of 211 nm exhibited better drug release and efficacy than larger aggregates, highlighting how size reduction can enhance solubility and bioavailability [86]. Size also affects cellular internalization mechanisms, with smaller particles often entering cells via energy-dependent pathways more effectively [122]. Zeta potential plays a crucial role in colloidal stability and electrostatic interactions with negatively charged bacterial membranes; positively charged (cationic) surfaces enhance adhesion, disrupting membranes and promoting payload internalization, as seen with chitosan-based carriers like alginate-chitosan (ALL@CS), which increase positive charge in acidic conditions due to protonation of amino groups [123]. A high absolute zeta potential value ( >|30| mV) usually indicates good physical stability by preventing aggregation through electrostatic repulsion, demonstrated by the stable SDS-zerumbone system (− 30.9 mV) in contrast to the unstable HPMC-stabilized formulation (− 3.37 mV) [124]. The PDI reflects size distribution homogeneity, where values below 0.3 indicate a monodisperse nanoparticle population essential for consistent pharmacokinetics and therapeutic efficacy, whereas higher values indicate size heterogeneity associated with variable biological responses and diminished effectiveness. Thus, the strategic design of nano-delivery systems for herbal antibacterials requires meticulous optimization of these physicochemical parameters to enhance targeting, uptake, and antimicrobial activity [125].

### In vitro and in vivo results

Described in Table 1, the use of nano delivery strategies for herbal medicines has achieved fascinating results given several in vitro and in vivo experiments to evaluate their antibacterial activity. Showing the antibacterial properties of these medications, zone of inhibition assay and MIC assessments are the most widely used methods of evaluation. Allicin from garlic, delivered via nanoparticle carriers (chitosan-lecithin and polydopamine/tannic acid-allicin@chitosan), and Honey Bee Propolis Extract (HBP) in a biodegradable hydrogel, both demonstrated broad-spectrum antibacterial activity against gram-positive, gram-negative, fungal, and yeast species.

Present as a nanosuspension, zerumbone from ginger displayed considerable antibacterial effect against *S. choleraesuis*. Delivered in many forms, curcumin demonstrated strong antibacterial effect against several gram-positive bacteria as well as against a gram-negative strain, *E. coli*. Broad-ranging antibacterial action of oregano-derived compounds, thymol and carvacrol, exhibited significant antibacterial capacity against both gram-positive and gram-negative bacteria. Delivered in numerous forms, carvacrol demonstrated amazing antibacterial action against several strains; co-delivery of other compounds through different nanocarriers enhanced their antibacterial properties. Aiming at a broad spectrum of bacterial strains, the complete in vitro data collectively show the efficacy of herbal components provided via nanocarriers to be potent antibacterial agents. Still, more in vivo study using repeated cell lines and testing is needed to confirm these positive in vitro results and so provide a more whole knowledge of their antibacterial characteristics.

## Anticancer applications

Second only to heart disease (Hossen et al., 2019; Prabhu et al., 2015), cancer is a complicated collection of disorders marked by aberrant cell growth and proliferation ranked as one of the main causes of mortality worldwide [126, 127]. Although traditional chemotherapy targets fast dividing cancer cells well, its therapeutic potential is limited and it sometimes lacks specificity, therefore damaging healthy tissues [127, 128]. Effective in treating many cancers, synthetic drugs including cabazitaxel [129], mitomycin c [130], and daunorubicin [131] can have side effects similar to traditional chemotherapy including bone marrow suppression and gastrointestinal disturbances. Given these difficulties, scientists are looking more and more for new anticancer agents in natural therapies and herbal medications.

Such molecules have long been important in traditional medicine and have been thoroughly studied for their anticancer action. Actually, around 60% of pharmaceutical anticancer drugs come from natural sources [132]. Investigating combined effects of herbal substances and nanocarriers in cancer treatment is a growing field of study. Because of their special physicochemical characteristics, nanocarriers present a good basis for targeted drug delivery, hence improving the efficacy and lowering the systemic toxicity of anticancer drugs. We explore in this part the increasing amount of research on the anticancer properties of herbal substances combined with different nanocarriers. We review common herbal medicines and nanocarriers, offering striking illustrations from current studies.

### Anticancer herbal compounds

For anticancer uses, we selected a panel of herbal compounds based on (i) strong preclinical evidence of antitumor effects observed across several relevant cancer models, (ii) successful incorporation in a nano-based delivery system with evidence of increases in solubility, stability, targeting, or biological activity, and (iii) chemical diversity, including flavonoids (quercetin, baicalein), stilbenes (resveratrol), catechins (EGCG), triterpenoids (ursolic acid), and alkaloids (berberine) [133, 134]. The examples selected allowed the review to discuss how to develop nanoformulations strategies based on distinct compound classes and types of cancers. Furthermore, a number of those agents (example-curcumin, berberine) even have dual antimicrobial and anticancer activity, reinforcing their potential importance to innovating in nano-delivery[135].

#### Turmeric

A polyphenol derived from the rhizome of turmeric (*Curcuma longa*), curcumin is a flexible herbal medicine valued for its many therapeutic properties, including anti-inflammatory, antioxidant, and anticancer effects. In the field of anticancer research, curcumin shows its amazing power by altering many signalling pathways important in cell growth, death, invasion, metastases, and angiogenesis. Among its molecular targets are STAT3, AKT, MAPK, p53, Bcl-2, COX-2, and nuclear factor-kappa B (NF-κB) [136]. The hydrophobic character of curcumin and low solubility in water (11 ng/mL) [137] or physiological fluids combined with its instability in neutral and basic environments have driven the development of strategies to improve its solubility and bioavailability [138] (Table 2).

**Table 2:** Summary of studies in the existing literature utilizing nano-delivery systems for herbal compounds for anticancer application

Name of the compound	Bioactive compound	Nanodelivery method	Fabrication of the carrier	In vitro and in vivo assays	Cell lines used	Refs.
Garlic	Allicin	CS-FA-PEG-lecithin NPs	Self-assembling procedure	MTT for viability assay, BTS and DPPH, etc	Colon cancer—HT-29	[75]
Turmeric	Curcumin	CMC and PVP deposited on graphene oxide	Layer-by-layer technique	Cell toxicity assay, Immunohistochemistry and immunofluorescence analyses—In vivo on 4T1 bearing breast cancer	Saos2 and MCF7 cell lines	[137]
	Curcumin	Curcumin-encased hydroxyapatite nanoparticles	Sol–gel method	Cell toxicity assay (MTT)	HeLa cell line	[175]
	Curcumin	NaCas (Sodium Caseinate)@CaP (Calcium Phosphate)	Rehydrating NaCas	CLSM images and cancer-cell-proliferation assay	A549 cancer cells	[139]
	Curcumin	Chitosan/chondroitin sulfate (CHT/CS) curcumin-charged hydrogels	Polyelectrolytic complexation	Apoptosis analysis—Colorimetric MTT assay	HeLa, HT29 and PC3 cancer cells—healthy (Vero)	[138]
	Curcumin	Curcumin-PVA/CNCs	Solution-casting method	In vitro cytotoxicity	Breast and liver cancer cells: MCF-7 and Huh-7 cells	[140]
	Curcumin	Co-delivery of curcumin and doxorubicin (Dox) with albumin nanoparticles		MTT assay, FACS measurements, co-localization of drugs within the lysosomes, The measurements of lysosomal pH, etc	MCF-7	[141]
	Curcumin	Iron oxide (Fe3O4) nanoparticles, grafted with hyperbranched polyglycerol (HPG) and FA	Polyol method	MTT assay and MRI Experiment	HeLa cells and mouse L929 fibroblasts	[176]
	Curcumin derivative C210	Conjugates of C210 and oleyl alcohol (OA)	Nanoprecipitation method	MTT assay, Animal Studies, Pharmacokinetic, etc	MCF-7 cells, 4T1 cells, MCF-10A cells and H22 cells	[177]
	Curcumin	Curcumin (Cur) loaded folic acid–chitosan conjugate (FC)Iron-based MOF, MIL-88B	Hydrothermal method	Decrease in tumor size;high uptake	Subcutaneously M109 cells(2 × 106) in Balb/C mice	[178]
	Curcumin	folic acid-conjugated curcumin-loaded nanoscale IRMOF-3	Two steps ultra-sonication methods	Tumor volumesupression; tumor weight decrease	Triple negativetumor-bearing BALB/Cmodel	[179]
Resveratrol	Resveratrol	Resveratrol loaded cationic liposomes	A thin film hydration method,	In vitro cytotoxicity studies, confocal images, Pharmacokinetic, etc.	HepG2 cells	[165]
	Resveratrol	Nanomicelles	Novel amphiphilic bioconjugate	MTT assay, oral pharmacokinetics study in Sprague Dawley rats	HCT	[145]
	Resveratrol	Co-delivery of resveratrol and quercetin with Zein and carboxymethyl cellulose (CMC)	Antisolvent precipitation	ABTS + and DPPH radical scavenging activity—Antioxidant activity evaluation	–	[180]
	Resveratrol	PEGylated liposome	Thin-Film Method	Increase the bioavailability of the drugs in vivo	BALB/c nude mice	[181]
Genistein	Genistein	lipid nanocore-protein nanoparticles grafted with ATRA and genistein	Nanoprecipitation method	MTT Assay, Cellular Internalization, In Vivo Study, in vitro: ELISA, Histopathological and Immunohistochemical Analysis	A549 lung cancer cells	[146]
	Genistein	Iron oxide nanoparticles decorated with carboxymethylated chitosan		MTT assay, Flow cytometry	MOLT-4, MOLT17 and Jurket cell lines	[182]
	Genistein	Co-delivery of Genistein (Gen) and Doxorubicin (Dox) with lipo-polymeric nanoconstructs	Film hydration	MTT and LDH assays, Receptor blocking study, apoptosis study, Matrigel transwell invasion assay, In vivo Matrigel plug assay, In vivo imaging and biodistribution study, Antitumor efficacy study in vivo, etc	MDA-MB-231	[147]
	Genistein	Casein-CFNPCalcium ferrite nanoparticles	The sol–gel method	In vitro MTT assay,	SKOV-3 MDA-MB-231 cancer cells, L929	[183]
	Genistein	Genistein–gold nanoparticles conjugates	A dual role of Gen	MTT test in vitro*, AnnexinV-FITC/PI staining and caspase detection, *etc	PC3, DU 145, LNCaP, and non-cancerous MRC-5 cells	[184]
	Genistein	BNC modified with cetyltrimethylammonium		IC_50_ for Free Genistein	SW480, SW620 and HaCaT	[185]
	Genistein	Surface modified genistein phytosome	Solvent evaporation method	A significant lowering the tumor size and tumor biomarkers (CEA, and CA)	Female albino mice	[186]
Baicalein	Baicalein	Baicalin-loaded PLGA nanoparticles	Ultrasonic double-emulsion	Cytotoxicity studies (flowcytometry), in vivo and ex vivo imaging, anti-tumor activity assay, etc	In vivo: C57BL/6 mice—Melanoma cells (B16)	[187]
	Baicalein	Nanofiber Scaffold Containing Hyaluronic Acid and Polyvinyl Alcohol	Electrospinning method was used in order to produce nanofibers	–	–	[150]
	Baicalein	Nanocomposites (BA@ZIF-8-PDA-PEG, BZPP)	One-step synthesis	Hemolysis test, MTT assay, live and dead staining assay	A549 cells	[149]
	Baicalein	Baicalein-loaded mPEG-PLGA nanoparticles	–	Cell Counting Kit-8(CCK8), BD C6 flow cytometry (FACS), Cell Uptake, Endocytosis pathway, qPCR, In vivo distribution, Therapeutic study, etc	NIH3T3 and murine breast cancer cell line	[188]
	Baicalein	Hyaluronic acid (HA)-lysine (L)-baicalein (BCL) self-assembled nanoparticles	Nanoprecipitation method	High anticancer efficiency and low systemic toxicity	Kunming mice	[189]
Quercetin	Quercetin	Co-delivery of quercetin and gefitinib with polyvinylpyrrolidone (PVP)-functionalized graphene oxide	Graphene oxide was modified with PVP in a carbodiimide-activated esterification reaction	In vitro cell viability assays, SRB assay, Phase contrast microscopic image	PA-1 and IOSE-364	[190]
	Quercetin	Co-delivery of quercetin (QU) and paclitaxel (PTX) with polyethyleneimine-Tocopherol Hydrogen Succinate/Hyaluronic Acid-Quercetin	Self-assemble into micelles	In vitro cytotoxicity, in vivo antitumor efficacy	MDA-MB-231/MDR1	[191]
	Quercetin	Membrane-camouflaged quercetin-melanin	Natural melanin was used to synthesize carrier PDA nanoparticles	MTT assay, RTCA, Flow cytometery, etc	HeLa, SGC-7901, and A549	[192]
	Quercetin	CuO nanorods	The microwave irradiation method through a chemical reaction	MTT, MIC	Regular human keratinocytes (HaCaT) and T-47D and MCF-7	[193]
	Quercetin	pH-sensitive—gelatin (G)-polyvinylpyrrolidone (PVP) coated graphene oxide loaded with quercetin	Hummer's method for synthesis of graphene oxide	MTT assay and flow cytometry	MCF-7 cell line	[151]
	Quercetin	hydrogel nanocomposite of chitosan (CS), halloysite, and graphitic-carbon nitride (g-C3N4)	Emulsification process	MTT assay	MCF-7 cell line	[194]
	Quercetin	Magnetite NPs (in combination with irradiation)	Biological simple nanoprecipitation	No tremor, convulsion, salivation, diarrhea, lethargy, sleepiness, or coma-like symptoms	Female white albino rats	[195]
Oldenlandia diffusa	Ursolic Acid	Polymer micelles	Thin-film dispersion method	In vivo toxicity, MTT assay	HepG2 and L-02	[152]
	Ursolic acid	paclitaxel (PUA-NPs@PTX)	PUA can self-assemble into nanoparticles	In vitro: Flow cytometry (BD FACSCalibur), MTT assay, Cell Cycle Analysis,—in vivo results in CT26 tumor-bearing mice for Biodistribution Study and Antitumor Efficacy, etc	NIH 3T3 and CT26 cells	[156]
	Ursolic acid	Chitosan modified PLA nanoparticles	Emulsion-solvent evaporation technique	In vitro release assay, Mucoadhesion assay, Hemocompatibility assay, MTT assay, pharmacokinetic study in vivo	B16-F10 and HEp-2—adult male Wistar rats	[196]
	Ursolic acid	Carboxymethyl chitosan nano-drug carrier modified by FA	Nanoprecipitation method	In vitro cell cytotoxicity with CCK-8 assay, In vivo antitumor efficiency, etc	MCF-7 and 4T1 cells—Female C57BL/6 mice	[197]
	Ursolic acid	Co-delivery of Bmi1 small interfering RNA with ursolic acid with folate receptor-targeted cationic liposomes	Thin film hydration method	Real-time PCR, MTT assay, In serum stability of FA-UA/siRNA-L, Anchorage-independent growth assay, etc	KB cells	[198]
	Ursolic acid	Nanophytoliposomes, coated with poly-L-lysine (PLL) and HA	Ethanol injection method	In vitro antitumor studies via in vitro (MTT) assay and apoptotic activity with flow-cytometry, in vivo tumorstudies, etc	SCC-7 and BT-474 cells, BALB/c nude mice	[199]
	Ursolic Acid	UA-liposomes	Thin film hydration method	flow cytometery, in vivo PK studies, C57BL/6 mice—tumorand growth inhibition studies	4T1, A549, PK studies, C57BL/6 mice	[200]
		UA polymeric micelles	Solvent evaporation method	MTT assay, Pharmacokinetics and biodistribution using SD rats, In vivo antitumor efficiency on mice	MG-63 cell line, NIH-3T3, and MG-63 cancer xenograft mice	[201]
Epigallocatechin gallate (EGCG)	Epigallocatechin gallate (EGCG)	Bombesin conjugated to EGCG loaded SLNPs	Double emulsification-evaporation method	*In-vitro* cytotoxicity (MTT assay), Cellular uptake studies, Migration assay, etc	C57/BL6 mice, MDA-MB-231 and B16F10 cells	[202]
	Epigallocatechin gallate (EGCG)	Gold nanoparticles (GNPs)	Reduction methods	Cell viability studies, Laminin receptor blocking study, PE Annexin V apoptosis detection, NF-κB transcriptional activity assay	MCF-10A, HPNE, RWPE1, MDA-MB-231, MIA PaCa, and PC3	[203]
	Epigallocatechin gallate (EGCG)	FA-functionalized nanostructured lipid carriers	High shear homogenisation and ultra sonication technique	MTT Caco-2 cell viability assay, etc	Caco-2 cell	[204]
	Epigallocatechin gallate (EGCG)	PLGA NPs	Emulsion solvent evaporation technique	The cellular uptake, cytotoxic activity, etc	Lung cancer cell lines A549 and H1299	[205]
	Epigallocatechin gallate (EGCG)	Gold nanoparticles (AuNPs)	Reducing gold salt with chitosan, functionalizing AuNPs with cysteamine using the Turkevitch method	In vitro toxic effect using Sulforhodamine B (SRB) method, In Vitro Release Studies, Cayman’s antioxidant assay kit, Caspase-3 Activation Assay	Human pancreatic cancer cell lines (BxPC3)	[206]
	Epigallocatechin gallate (EGCG)	Co-delivery of EGCG and rutin with PEG-PCL diblock copolymer	Self-assembly with slight modification	In vitro drug release analysis, MTT assay, caspase-9 and -3 activity, intracellular ROS measurement, Cellular uptake assay, Hemolysis assay	A549 and HeLa human cancer	[158]
Berberine	Berberine	Carbon nanoparticle—C60 fullerene	Ultrasonication method	Flow Cytometry, Fluorescent Microscopy, etc	CCRF-CEM (ACC 240)	[161]
	Berberine	Lyotropic liquidcrystalline nanoparticles (LCNs)	Ultrasonication method	MTT assay, accumulation in both cell lines, Flow cytometry analysis, cell cycle analysis, PI staining, etc	MCF7 and Caco-2	[207]
	Berberine	Gold nanoparticle-collagen	Patented method according to previous study	MTT assay, LysoTracker Assay, Gelatin Zymography Analysis, Assessment of Cell Migration Ability, in vivo study Tumor Xenograft Mouse Model, etc	A549, Colo-205, BAEC and Her-2—male BALB/c nude mice	[208]
	Berberine	Bovine Serum Albumin nanoparticles	Desolvation method	MTT assay, Trypan Blue Assay, Apoptosis study with fluorescence microscope, Cellular uptake study	MDA-MB-231	[209]
	Berberine	Co-delivery of Curcumin and berberine with Zein-Chitosan nanoparticles	Anti-solvent precipitation method	In vitro release study, MTT assay, Cellular uptake, Apoptosis analysis with Ethidium bromide (EB) and Acridine orange (AO) satining, ELISA	MDA-MB-231, Human A549 lung cancer, human foreskin fibroblast	[210]
	Berberine	EDC-crosslinked BSA	The desolvation method	In vitro drug release study, Blood compatibility experiments by Hemolysis assay and Haemagglutination assay, Migration inhibition assay, Chromatin condensation assay with DAPI fluorescent dye, etc	Glioblastoma (LN229) cell line	[160]
Artemisinin	Artemisinin	Artemisinin-loaded liposomes modified with reversibly activatable cell-penetrating peptide	Thin film hydration method	In vitro cytotoxicity study by MTT assay, In vitro cellular uptake studies by Flow cytometry analysis, Injection irritation, in vivo studies for biodistribution, pharmacokinetic study, antitumor efficacy study, etc	4T1, Female Balb/C mice	[167]
	*Artemisia vulgaris* L. essential oil	PLGA-chitosan-FA nanoparticle	Single emulsion solvent evaporation method	MTT assay, Flow cytometry, etc	HT-29 cells and HFF	[211]
	Artemisinin	Co-delivery of Artemisinin and Chrysin with PEGylated PLGA nanoparticles	Double emulsion (W/O/W) method	In vitro evaluation drug release, MTT-based cytotoxicity assay, etc	T47D human breast cell line	[212]
	Artemisinin	Folate-modified erythrocyte membrane nanoparticles loaded with iron oxide and artemisinin	The liposome extrusion method	In vitro and in vivo biocompatibility studies via the CCK-8 assay, In vitro and in vivo targeting studies, confocal laser scanning microscope, in vivo antitumor effect, etc	4T1 and RAW264.7 cell line—female Balb/cmice	[166]
	Artemisinin	Co-delivery of Artemisinin (Art) and Cur with niosomal nanoparticles		In vitro drug release, MTT, qRT-PCR	SW480 cells	[164]
Ginsenoside Rg3	Ginsenoside Rg3	Co-delivery of Paclitaxel with multifunctional ginsenoside Rg3-based liposomal system (Rg3-LPs)	Thin-film hydration method	Molecular docking study for Ginsenoside Rg3, Cellular uptake assay, Penetration of C6 tumor spheroids, In vitro BBB model crossing, In vitro cytotoxicity and apoptosis by MTT and Annexin V-FITC/PI staining, harmacokinetic, biodistribution, and anti tumorstudies, etc	C6 murine glioma cells, BCECs and HUVECs, Balb/c nude mice, Pharmacokinetic and biodistribution in male ICR mice, C6-glioma rats	[169]
	Ginsenoside Rg3	Graphene Oxide Nanoparticle—linked with photosensitizer indocyanine green, FA and PEG	Hummers and Offman’s method,	In vitro drug release, CCK-8 assay for cell viability,flowcytometry of CD117- or Stro-1-positive osteosarcoma cell, in vivo tumoranalysis in Balb/c nude mice, etc	The MG63 and U2OS cells, Balb/c nude mice	[174]

Among these methods, nanoparticulate drug delivery systems have become well-known for their ability to raise the water dispersibility of hydrophobic molecules such as curcumin. Characterized by their nano-size and core–shell structure, one creative technique uses sodium caseinate@CaP (calcium phosphate) (NaCas@CaP) nanodelivery systems (Table 2) [139]. These systems significantly enhance the stability of encapsulated curcumin, offering pH-responsive release in proximity to cancer cells, thereby augmenting its cellular antioxidant and anticancer efficacy.

Another promising avenue employs curcumin-loaded polyvinyl alcohol/cellulose nanocrystals (PVA/CNCs) membranes as localized delivery systems for breast and liver cancer (Table 2) [140]. These membranes are possible anti-infective biomaterials for wound healing in breast and liver cancer cases since they show broad-spectrum antimicrobial activity and can thus restrict microbial growth by over 96–99%. Moreover, creative approaches have been used to solve the problem of adaptive treatment tolerance in cancer treatment, a phenomena wherein cancer cells develop resistance to therapeutic medications over time [141] (Table 2). These efforts show great progress in using curcumin's anticancer power in the dynamic terrain of nano-delivery devices.

#### Resveratrol

Renowned stilbenoid naturally present in grapes, red wine, peanuts, and berries, resveratrol has become a powerful herbal medicine with trifecta of therapeutic effects: antioxidant, anti-inflammatory, and anticancer. Regarding anticancer effects, the several modes of action of resveratrol have attracted much research. By means of CD95 signalling activation and elevation of CD95L expression, resveratrol has the ability to cause death in cancer cells. Targeted cancer treatment may find a bright future in this natural capacity to induce programmed cell death. Resveratrol also shows great ability to stop angiogenesis and tumour formation, mostly because of its modulation of matrix metalloproteinases (MMPs) and vascular endothelial growth factor (VEGF) [136]. Resveratrol is a strong enemy against cancer since it reduces the development of new blood vessels and disturbs the milieu necessary for cancer progression. Apart from directly affecting cancer cells and tumour microenvironment (TME), resveratrol shows anti-inflammatory action by preventing the creation of MMPs and pro-inflammatory cytokines [142]. These activities demonstrate resveratrol's multifarious approach in preventing the disease and aid to reduce the inflammatory cascade linked with cancer growth and progression. Resveratrol's flexible modes of action include autophagy induction, death induction, angiogenesis inhibition, metastases inhibition, and cancer cell metabolism reorientation [143]. Such variation in its anticancer properties emphasizes its possible use as a multifarious agent in the battle against several cancer types, including lung cancer and prostate cancer. Furthermore, recent developments have included creative resveratrol delivery techniques like transpapillary distribution for breast cancer treatment [144]. Emphasizing the need of customized delivery systems to maximize the healing capacity of this strong herbal ingredient, inulin-pluronic-stearic acid-based double-folded nanomicelles have also been presented for pH-responsive resveratrol distribution [145] (Table 2).

#### Genistein

Strong isoflavone naturally present in soybeans and some legumes, genistein has attracted a lot of interest for its several anticancer properties, including anti-inflammatory and antioxidant ones. It is a strong anticancer agent since it shows amazing ability to block tyrosine kinases fundamental in cell growth and survival. Furthermore, genistein affects cancer cells by causing cell cycle arrest in G2/M phase and death, coordinated by control of major actors like cyclin B1, CDK1, p21, p27, Bax, and caspase-3 [136]. Combining all-trans retinoic acid (ATRA), which causes cell cycle arrest at the G1 phase and cell death via nuclear retinoic acid receptors, with genistein, a potent tyrosine kinase inhibitor, increases cytotoxic effects and cellular absorption in A549 lung cancer cells. This combination therapy shows promise for lung cancer treatment. For the co-delivery of genistein and ATRA to lung cancer cells, one such new method is the fabrication of inhalable dry powder nanocomposites of dual-targeted hybrid lipid-zein nanoparticles [146] (Table 2). Furthermore, by use of the HSPG receptor, genistein has shown its mastery in receptor-mediated endocytosis in human breast cancer cells (MDA-MB-231) [147] (Table 2). This concentrated approach holds considerable promise for the focused distribution of genistein to breast cancer cells, therefore enhancing its therapeutic activity. But low oral bioavailability and restricted water solubility of genistein make clinical application difficult. Techniques to overcome these constraints will enable to maximize its biodistribution and absorption in cancer treatment.

#### Baicalein

**Fig. 3:**
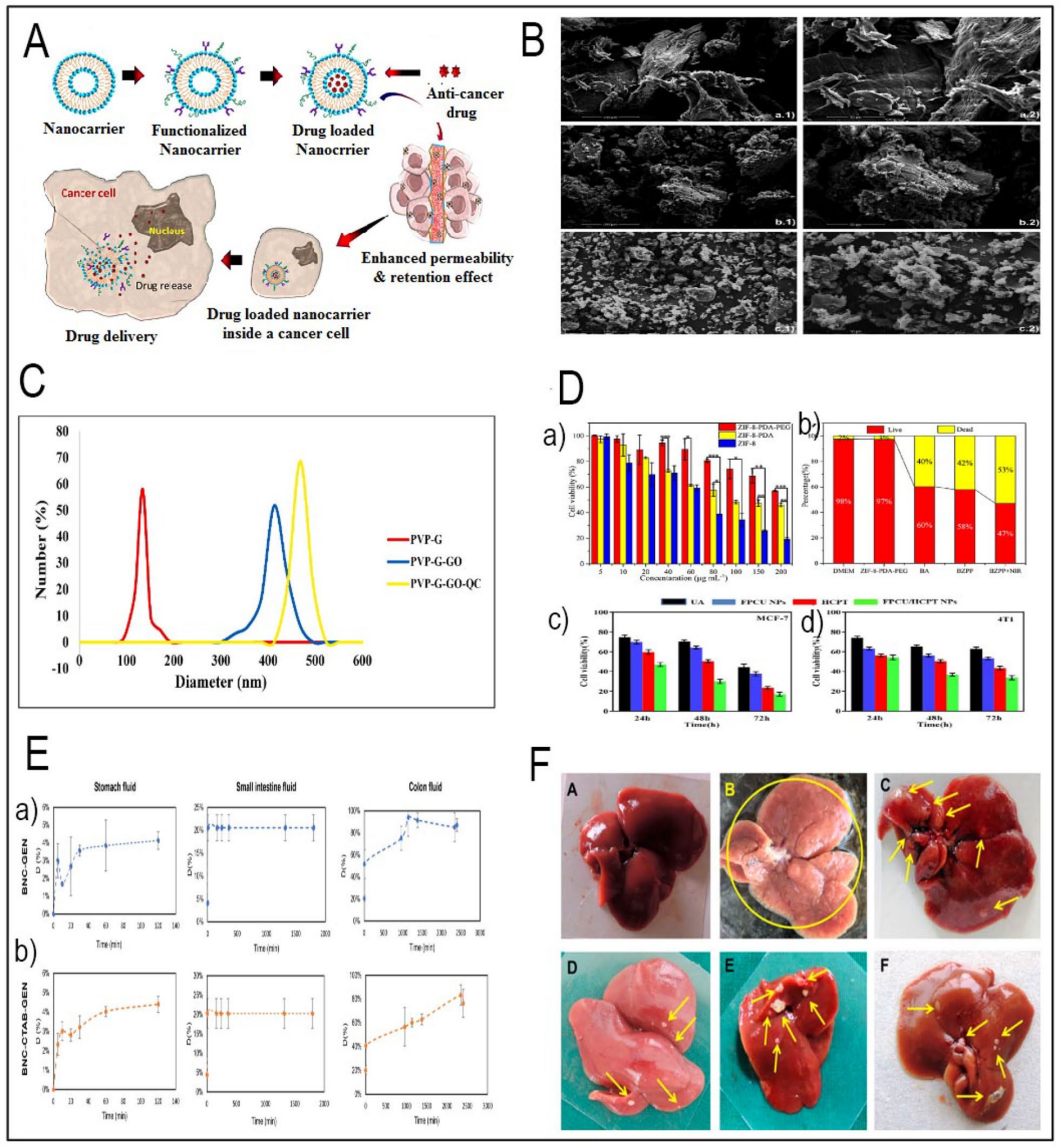
**A** Illustration of nanocarrier targeting, binding, internalization, and drug release in cancer therapy (Redesigned from reference [127] with permission). **B** SEM images of PEC, PEC-CUR and PEC-T-CUR. Scale bars of 100 μm (images in the left, magnification ×1000) and 50 μm (images in the right, magnification ×2000) (Redesigned from reference [138] with permission). **C** DLS analysis and the size distribution of PVP-G, PVP-G-O and PVP-GGO-QC nanocomposite (Redesigned from reference [151] with permission). **D **(**a**) Cell viability of ZIF-8, ZIF-8-PDA, ZIF-8-PDA-PEG and A549 cells after incubation for 24 h (Redesigned from reference [149] with permission). (**b**) Survival area of A549 cells treated with DMEM, ZIF-8-PDAPEG, free BA, BZPP, BZPP + NIR for 24 h (BA concentration: 20 μgmL^−1^) (Redesigned from reference [149] with permission). (**c**) Cell viability of MCF-7 and (**d**) 4T1 cells dealt with different groups was detected by CCK-8 assay (Redesigned from reference [197] with permission). **E **(**a**, **b**) The release profile of genistein within gastrointestinal fluids (Redesigned from reference [185] with permission). **F** Representative illustrations of rat liver specimens: **A** the typical group of liver tissue exhibits a normal appearance, showing no macroscopically detectable pathological alterations. **B** The disease control group's liver tissues exhibited notable alterations in terms of hue, texture, and consistency. The tissue displayed a pallid pink coloration along with enlarged dimensions and a wrinkled surface adorned with numerous visible nodules (indicated by yellow arrows). **C** Prophylactic RS treated group demonstrated very few nodules (yellow arrows) and lesions. **D** Prophylactic RL5 treated group in rats showed marked reduction in the number of nodules and damaged caused by NDEA. **E**,** F** Therapeutic RS and RL5 treated groups in rats showed reduction in the number of nodules (Redesigned from reference [165] with permission). (p-value = p, *p < 0.05, **p < 0.01, *** p < 0.001)

Baicalein is a flavonoid derived from the root of *Scutellaria baicalensis Georgi*, a Chinese herb with diverse medicinal benefits, such as antibacterial, antioxidant, antiviral, and anti-inflammatory effects. Among its many virtues, baicalein’s anticancer activity is especially remarkable, as it can modulate the cell cycle, scavenge oxidative free radicals, induce apoptosis, and suppress tumor infiltration and spread. By releasing cytochrome c into the cytoplasm and activating caspase-9, Baicalein causes death in cancer cells, therefore triggering a planned biological response against malignancy [136]. Baicalein thus shows promise as a possible future osteosarcoma candidate. Moreover, baicalein's anticancer ability reaches the domain of angiogenesis control by downregulating important elements including matrix metalloproteinase-9 [148]. Stressing cancer cell survival as well as the building of new blood vessels required for tumor progression, this dual-action approach highlights its possible anticancer activity. Gao et al., (2021) coupled chemotherapy with photothermal therapy using nanocomposites based on ZIF-8 to produce a pH-responsive drug delivery system known baicalein @zeolite imidazolate frameworks-8 (ZIF-8)-polydopamine (PDA) (BZPP) [149]. MTT test evaluations of drug carrier cytotoxicity Fig. 3D (a clearly indicates a dose-dependent relationship whereby the viability of A549 cells lowers gradually with increasing nanocarrier concentration. In the framework of cancer treatment, the challenge usually goes beyond the eradication of tumor tissue following surgery. The cell survival rates of the ZIF-8-PDA-PEG (Zeolitic Imidazolate Framework-8, Polydopamine, and Polyethylene Glycol), BZPP (Buffer Zone Protection Program), and BZPP + near-infrared (NIR) groups displayed in Fig. 3D part b [149]. Clinically, remaining cancer cells and the required means of rebuilding damaged bone structure after surgery raise challenging concerns. Functional biomaterial scaffolds provide promising responses linking damaged tissues and stopping cancer recurrence [148]. Using electrospinning, researchers recently explored the possibility of baicalein to mix it with a hyaluronic acid-polyethylene oxide-transforming growth factor beta-2-polyvinyl alcohol nanofiber scaffold (Table 2) [150]. Since it showed that baicalein-loaded nanofiber scaffolds may sufficiently prevent local recurrence of bone cancers, this new approach underlined the tremendous adaptability of these materials as biomedical tools in the fight against cancer.

#### Quercetin

Quercetin, a flavonoid abundantly found in various fruits and vegetables, possesses a versatile repertoire of healing attributes, encompassing antioxidant, anti-inflammatory, and anticancer effects. Among its remarkable anticancer attributes, quercetin's ability to induce apoptosis in cancer cells takes centre stage. It does so by orchestrating a complex interplay involving intrinsic and extrinsic pathways, featuring key players like Bcl-2 family proteins, cytochrome c, caspases, FasL, and TRAIL. Additionally, quercetin exerts control over cell growth by regulating the PI3K/AKT/mTOR signalling cascade, further bolstering its anticancer potential [136]. In a separate study, Najafabadi et al., employed nanocarriers consisting of gelatin (G)-polyvinylpyrrolidone (PVP) coated graphene oxide (GO) for the first time. These nanocarriers were loaded with the drug quercetin (QC), and a dual nanoemulsion water/oil/water system with bitter almond oil was developed as a membrane to regulate the release of the drug. The size distribution of the nanocarriers was ascertained using dynamic light scattering (DLS) analysis. The results are displayed in Fig. 3C. The study's findings indicate that PVP-G-GO-QC holds promise as a potentially innovative approach for cancer treatment [151].

#### Ursolic acid

Ursolic acid (UA), also known as urson, prunol, or malol, is a pentacyclic triterpenoid compound widely found in nature. This leads to DNA damage and cell cycle arrest at the G2/M phase. This immune-boosting aspect not only strengthens the body’s natural defences but also complements the herb’s anticancer arsenal [136]. Ursolic acid (UA), a compound widely distributed in various dietary plants such as *Oldenlandia diffusa,* has emerged as a promising anticancer agent. UA’s anticancer effects span across various aspects of cancer biology, making it a potent weapon against the disease [152] (Table 2). It modulates various cellular factors that regulate cell proliferation, metastasis, apoptosis, angiogenesis, and autophagy. Liver cancer, among other cancer types, has been a focal point of UA’s anticancer activities [153]. UA exerts its effects through diverse mechanisms, such as affecting NF-κB factors and apoptosis signalling pathways in tumor cells. However, UA’s hydrophobic nature has posed challenges to its clinical application [154, 155]. To overcome these limitations, an innovative method entails development of a polymeric drug delivery system known as poly (ursolic acid) (PUA), which is synthesized through the polycondensation of UA. PUA can self-assemble into nanoparticles (PUA-NPs), offering a dual benefit [156] (Table 2). It not only serves as a drug carrier but also introduces an additional therapeutic dimension. This approach unveils thrilling prospects for improved drug delivery and therapeutic effectiveness.

#### Epigallocatechin gallate

**Fig. 4:**
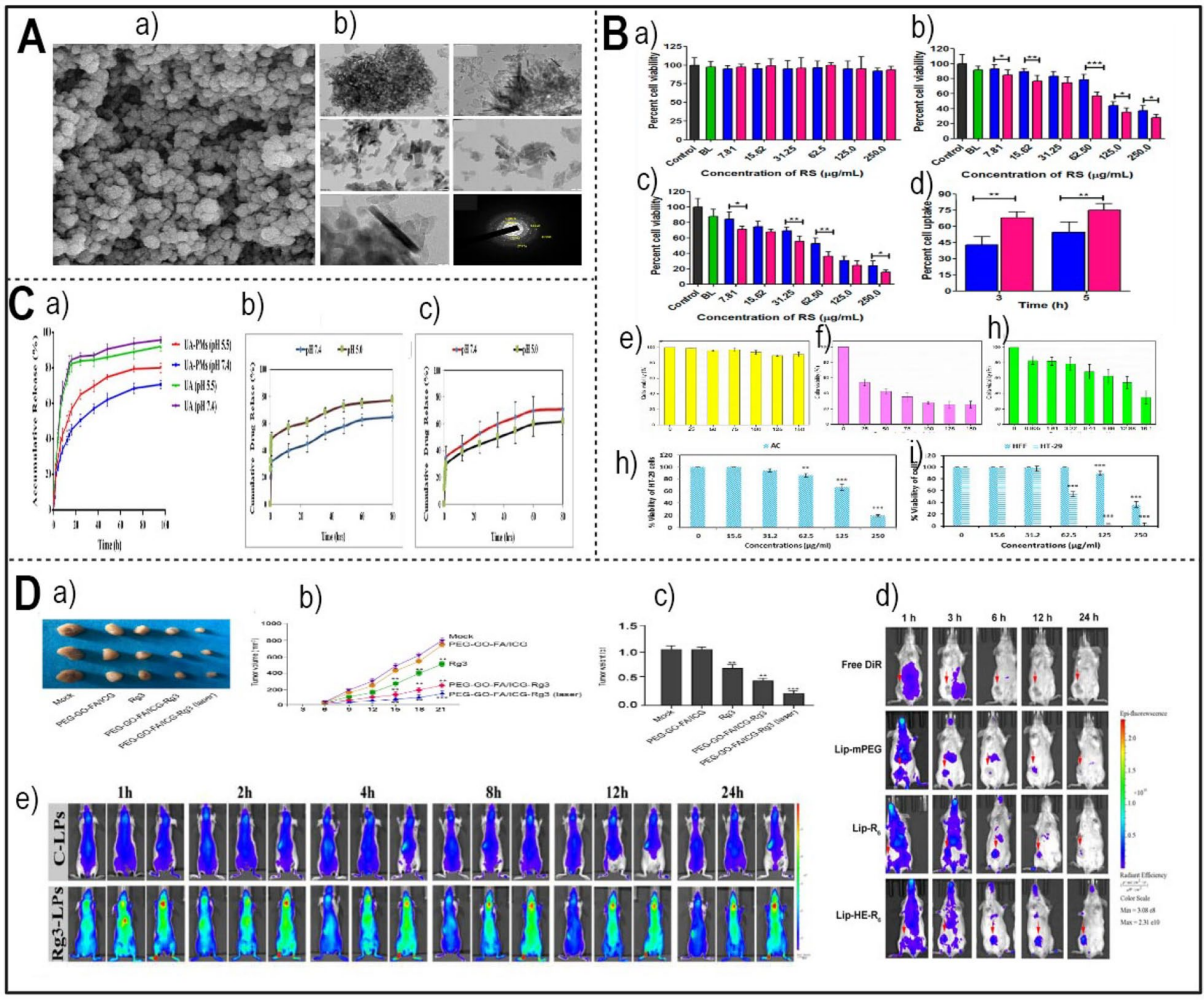
**A a)** FE-SEM images of Cur–Art NioNPs (Redesigned from reference [164] with permission). (**b**) TEM photos show the magnification levels of ×40000, ×40000X, ×80000, ×80000, ×200000, and a SAED pattern (Redesigned from reference [175] with permission). **B **(**a**) The cytocompatibility of RS and RL5 was assessed by measuring their cytotoxicity against normal mouse fibroblast cell lines (L929) using the colorimetric MTT test (Redesigned from reference [165] with permission). (**b**, **c**) The cytotoxicity of BL, RS, and RL5 on HepG2 cell lines was assessed over 24 and 48 h. Cell viability was measured using the MTT test at various drug doses (Redesigned from reference [165] with permission). (**d**) A bar graph illustrating the quantitative cell internalization of RS and RL5 in HepG2 cancer cells. The evaluation was conducted at 3 and 5 h time intervals using HPLC (Redesigned from reference [165] with permission). An in vitro cytotoxicity analysis of the BSA NPs (**e**), pure BER (**f**), and BER–BSA NPs (**g**) against the LN229 cell line following 24 h incubation (Redesigned from reference [160] with permission). (**h**) MTT assay. A Decreased HT-29 cell viability in AC treatment (Redesigned from reference [75] with permission). (**i**) MTT assay. comparison of growth inhibition of HT-29 cancer cells compared to HFF in AC-PLCF-NPs treatment (Redesigned from reference [75] with permission). **C **(**a**) The in vitro cumulative release profile of ursolic acid (UA) from polymeric micelles loaded with UA (UA-PMs) in a phosphate-buffered saline (PBS) solution at pH 7.4 or pH 5.5, and at a temperature of 37 °C, is compared to the release profile of free UA (Redesigned from reference [152] with permission). Drug release kinetics of (**b**) EGCG and (**c**) Rutin (Redesigned from reference [158] with permission). **D **(**a**) Representative images of dissected tumors from nude mice are presented (Redesigned from reference [174] with permission). (**b**) The average tumor volume is calculated and shown (Redesigned from reference [174] with permission). (**c**) The average tumor weight is calculated and shown (Redesigned from reference [174] with permission). (**d**) The in vivo imaging of 4 T1 tumor bearing mice after intravenous injection of free DiR, DiR loaded Lip-mPEG, Lip-R6, and Lip-HE-R6 (Redesigned from reference [167] with permission). (**e**) In vivo fluorescence imaging of C6 orthotopic glioma bearing nude mice treated with DiR-loaded Rg3-LPs at different time points (Redesigned from reference [169] with permission). (p-value = p, *p < 0.05, **p < 0.01, *** p < 0.001)

Epigallocatechin gallate (EGCG), a potent catechin in green tea, has remarkable anticancer properties. Green tea is one of the most widely consumed drinks globally and has many health advantages, mainly due to EGCG. EGCG has a multifaceted approach to combating cancer. This natural phenolic compound can target various cancer types by inducing cell cycle arrest, apoptosis, autophagy, and oxidative stress in tumor cells. These are key processes that EGCG can modulate to inhibit cancer growth [136]. EGCG also affects tumor progression and metastasis by targeting molecular factors such as VEGF, MMPs, and NF-κB. By doing so, EGCG can curb tumor angiogenesis, invasion, and metastasis, making it a comprehensive anticancer agent. However, EGCG has poor bioavailability, limiting its clinical. To address this challenge, nano formulations of EGCG are arising as a promising alternative. These nano chemo preventive approaches include various types of EGCG nanoparticles, such as lipid-based, polymer-based, carbohydrate-based, protein-based, and metal-based nanoparticles [157]. In a study conducted by Saha et al., an amphiphilic PEG-polycaprolactone (PCL) diblock copolymer was utilized. This copolymer was co-encapsulated with epigallocatechin-3-gallate and rutin nanorods, which had dimensions of approximately 110 nm in length and 26 nm in width. The purpose of this combination therapy was to synergistically combine the anticancer and antibacterial properties. The spectrophotometric approach was used to evaluate the release kinetics of EGCG and rutin from co-encapsulated nanorods. The experiments were conducted at 37 ºC in PBS with varying pH levels (7.4 and 5.0), as depicted in Fig. 4C part B. The study found that the release of rutin from co-encapsulated nanorods followed a slow and persistent pattern at various pH levels. Additionally, there were no noticeable variations in the release rates at different pH values (Fig. 4C part a [158]. This field holds the potential to unlock the full therapeutic power of EGCG and enhance its clinical utility. EGCG’s journey from a humble tea leaf to a potent anticancer agent shows the importance of exploring natural compounds in the fight against cancer. Innovative nanotechnological approaches are poised to revolutionize its clinical impact.

#### Berberine

Berberine, a potent isoquinoline alkaloid from the roots of medicinal plants such as goldenseal, barberry, and Oregon grape, has remarkable anticancer properties. Berberine has multiple mechanisms that target critical signalling pathways in cancer cells. Berberine can modulate key cellular factors, such as activated protein kinase, mitogen-activated protein kinases, and NF-κB. Berberine can intervene at multiple stages of tumorigenesis in cancerous cells [159]. These actions make berberine a versatile anticancer agent. In a separate study, scientists sought to create EDC-crosslinked BSA NPs as a method of delivering drugs by enclosing berberine (BER). The objective was to evaluate the effectiveness of the medication discharge in treating human glioblastoma LN229 cells. An MTT experiment was conducted to assess the cytotoxic effects (Fig. 4B part e, f,and g [160]. Researchers have also explored innovative delivery methods to optimize berberine’s therapeutic impact. One example is nanotechnology, where berberine is combined with nanosized carbon nanoparticles, such as C60 fullerene (C60) [161] (Table 2). This method enhances the delivery of berberine into leukemic cells, offering improved efficacy and precision in cancer treatment. Berberine’s evolution from botanical origins to a multifaceted anticancer agent shows the potential of nature’s pharmacopeia in the fight against cancer. Novel delivery methods may expand the clinical impact of berberine in cancer therapy.

#### Artemisinin

The plant *Artemisia annua* contains the sesquiterpene lactone artemisinin, which has antitumor characteristics. By producing ROS and stimulating the mitochondrial pathway, it has the ability to trigger both autophagy and apoptosis in cancerous cells [162]. Artemisinin also inhibits tumor angiogenesis, invasion, and metastasis by suppressing the expression of factors like VEGF and MMPs. These factors facilitate cancer’s growth and dissemination [163]. Researchers have developed novel strategies to optimize artemisinin’s anticancer effects. In another study, Amandi et al., employed curcumin–artemisinin (ART) co-loaded niosomal NPs (Cur–ART Nio NPs) as a therapeutic agent against colorectal cancer cells. The topography and shape of the generated Nio NPs were examined using field emission scanning electron microscopy (FE-SEM), as shown in Fig. 4A. The high-performance liquid chromatography (HPLC) measurement of the cell extract after 5 h revealed that Resveratrol (RS) and optimized liposomes formulation (RL5) had cellular internalization rates of 54.2% and 74.98%, respectively (Fig. 4A part a) [164, 165]. Another example is folate-modified erythrocyte membrane-camouflaged nanoparticles (Table 2) [166]. These nanoparticles enhance the delivery and impact of artemisinin in cancer therapy. They have components such as perfluorohexane, magnetic Fe_3_O_4_, and artemisinin that offer precision cancer treatment. As an additional illustration, Yu and colleagues employed a reversibly activatable cell-penetrating peptide to design liposomes loaded with artemisinin known as ART-Lip-HER6. The purpose was to target tumors, and the performance of these modified liposomes was examined both in vitro and in vivo. The targeting of different formulations to tumors was assessed in Balb/C mice with 4 T1 tumors. Figure 4D part d displays the distribution of DiR-loaded liposomes in animals at different periods [167]. Artemisinin’s transformation from a traditional remedy for malaria to a potent anticancer agent shows the potential of natural compounds in the fight against cancer. Innovative delivery methods may enhance its clinical impact.

#### Ginsenoside Rg3

Ginsenoside Rg3, a saponin from ginseng roots, has anticancer properties. It can inhibit cell proliferation, induce apoptosis, and stimulate autophagy in cancer cells. These are key processes that Ginsenoside Rg3 can modulate to suppress cancer growth [168]. Ginsenoside Rg3 also reverses multidrug resistance, a major challenge in cancer treatment. It restores the effectiveness of therapeutic drugs by blocking the mechanisms that make cancer cells resistant [169]. Ginsenoside Rg3 affects the tumor TME as well. It reduces angiogenesis, inflammation, and immune evasion, creating a less favourable environment for cancer to thrive. Moreover, Ginsenoside Rg3 inhibits cancer metastasis and recurrence, two factors that increase cancer’s lethality. It prevents the spread and resurgence of cancer, making it a formidable anticancer agent [170–173]. Furthermore, various types of nanoparticles were employed to transport Ginsenoside Rg3 to cancer cells. The nanoparticles were synthesized by Lu et al., (2021) utilizing GO connected to a photosensitizer called indocyanine green. FA and PEG were also incorporated into the nanoparticles, which were then embedded with Rg3. The researchers investigated the impact of the formulation with photodynamic therapy for the management of osteosarcoma. The study revealed that the advancement of tumor was significantly inhibited by Rg3. Furthermore, the formulation treatment and NIR laser showed an even stronger suppressive effect on tumor growth in nude mice. Figure 4D part a, b, and c indicate that this impact was detected in the system [174]. In another study, Zhu and colleagues created a versatile liposomal system based on ginsenoside Rg3, known as Rg3-LPs. The active glioma selectivity and intratumoral dispersion capacities of Rg3-LPs were significantly enhanced in vivo. Due to the fact that the C6 glioma cell line was derived from adult rats, there is a possibility that implanting C6 cells into balb/c mice of different strains could elicit an immunological response and perhaps impact the outcomes of immune regulation. Thus, a rat model with a C6 brain tumor was created to validate the pharmacodynamics of the liposomes (Fig. 4D part e) [169]. Ginsenoside Rg3’s transformation from ginseng roots to a promising anticancer agent shows the ability of natural substances in the fight against tumor. Research may reveal more of its mechanisms and impact.

### Delivery of herbal compounds in anticancer application

The delivery of anticancer herbal compounds is a burgeoning field with promising implications for cancer treatment and therapy. Novel techniques in nanoscale have been investigated as potential solutions to the problems caused by these chemicals' poor absorption, stability, and dispersion. This section delves into various nanocarriers and strategies used for the efficient delivery of anticancer herbal formulations, with a focus on their advantages and applications. Figure 3A illustrates the mechanism of action of a nanocarrier-based drug delivery system in cancer treatment.

#### Hydroxyapatite

Hydroxyapatite (HAp), a vital biological molecule found in bones, has emerged as a versatile material with a multitude of clinical applications. Sebastiammal et al., (2020) highlight its significance in significantly improving the bioactivity and compatibility of synthetic molecules. A comprehensive structural investigation of pure HAp was conducted. The Fig. 4A part b illustrates the various magnifications of pure surfactant-free HAp NPs, providing a global view. In the context of anticancer therapy, HAp has shown promise in serving as a carrier for herbal compounds, offering enhanced bioavailability and controlled release, thereby improving the therapeutic efficacy (Table 2) [175].

#### Hydrogels

Using hydrogel—a 3D structure made up of hydrophilic polymers—in conjunction with antitumor herbal compounds such as curcumin (CUR), presents an innovative approach to drug delivery. This strategy holds the potential to provide superior functionality to composite materials, particularly in medication transportation and nanomedicine applications. nanohydrogels offer benefit of protection of loaded drugs and controlled release through stimuli-responsive mechanisms or biodegradable bonds [118, 138].

Chondroitin sulfate (CS), natural polysaccharides, have garnered interest in the medical field due to their unique characteristics. When combined with CUR, they can form physical polyelectrolyte complex hydrogels that provide a good level of drug dispersion. This approach, as highlighted by Caldas et al. (2021), offers a potential platform for the controlled discharge of anticancer herbal formulations, ameliorating their curative capacity [138]. In their study, Caldas and colleagues synthesized CS and chondroitin sulfate hydrogels loaded with CUR. The hydrogels were then tested for their ability to induce apoptosis in cancerous cells. Figure 3B displays SEM images, revealing a noticeable reduction in particle dimension when compared [138].

#### Liposomes

Liposomes, among various nanoparticle formulations, have proven to be highly effective in delivering therapeutic drugs and imaging agents. Their structural properties closely mimic natural cellular biomembranes, offering advantages such as biocompatibility, enhanced stability, sustained drug release, biodegradability, safety, and scalability [165]. Cell viability was assessed using the MTT test at several doses of the medication (Fig. 4B part a–d). when resveratrol (RS) loaded cationic liposomes exhibited comparable cell viability, indicating their cytocompatibility. Two mice from the unaffected group who had the illness were taken apart after the ninth week of the trial, and histology was used to examine the specimens to confirm that malignancy had successfully developed. The liver specimens' morphology was evaluated for all groups, as shown in Fig. 3D part a–f. When RS and RL5 were given to rats with hepatocellular carcinoma, there were significantly fewer liver nodules when contrasted with the group of animals that had the condition [165]. emphasize the success of liposomal nanoscale formulations in anticancer drug delivery, underscoring their potential in improving treatment outcomes (Table 2) [165].

#### Bacterial nanocellulose

Bacterial nanocellulose (BNC) is a nontoxic substance with distinct structural characteristics, making it suitable for various medical applications. Castaño and colleagues (2022) examined the use of BNC and BNC that has been combined with cetyltrimethylammonium (BNC-CTAB) in order to assess their effectiveness as carriers for genistein, with the goal of achieving controlled release for cancer chemoprevention [185]. They discussed the potential of BNC in drug delivery systems. Furthermore, the percentage of genistein (GEN) dispersed from dehydrated films comprising BNC-GEN and BNC-CTAB-GEN was evaluated and is illustrated in Fig. 3E part a, b. BNC's three-dimensional network of nanoribbons, high surface area, open porosity, and tensile strength make it a promising candidate for encapsulating anticancer herbal compounds, offering controlled drug release and targeted therapy (Table 2) [185].

#### Polymer micelles

Polymer micelles (PMs), formed from amphiphilic block copolymers, have gained recognition as nano-sized medication formulation. Zhou et al., (2019) highlight their capacity to dissolve in water insoluble anticancer compounds within their hydrophobic inner core. The release kinetics of UA-PMs were assessed in vitro at a temperature of 37 °C. The releasing media used was PBS, and the pH settings tested were 7.4 and 5.5 (Fig. 4C part a). PMs offer a novel approach to enhance drug solubility and improve the delivery of herbal compounds, thereby augmenting their anticancer activity [152].

#### Carboxymethyl chitosan

Carboxymethyl chitosan (CMCS), characterized by its water solubility, biocompatibility, and biodegradability, functions well as a carrier for sustained medication transportation. Jing et al. (2021) emphasize the potential of CMCS in delivering anticancer herbal compounds, offering a controlled and sustained release, thereby enhancing the therapeutic effectiveness (Table 2) [197]. An optimal nano-drug carrier should exhibit unique responses to intricate biological surroundings, in addition to demonstrating high biocompatibility and minimal cytotoxicity. The survival rates of NPs following exposure to breast cancerous cells for durations of 24, 48, and 72 h, shown in Fig. 3D part c, d [197].

In conclusion, the delivery of anticancer herbal compounds through nanotechnology-based approaches offers exciting possibilities for improving cancer treatment. These innovative delivery systems, including hydroxyapatite, micro/nano-hydrogel, chitosan, liposomal nanoparticles, and bacterial nanocellulose, among others, hold the ability to improve the availability, stability, and targeting of herbal substances, ultimately contributing to more effective cancer therapy.

### In vitro and in vivo results

The utilization of nano delivery systems for herbal compounds has yielded encouraging outcomes in various assays. Among the methods reviewed in Table 2, in vitro tests (such as MTT and cell toxicity assays) were commonly applied to evaluate the effectiveness of nano-delivered herbal substances on different cell lines. Additionally, assays related to cytotoxicity, apoptosis, and antioxidant capacity were frequently conducted. Notably, the most frequently employed cell lines included cancer cell lines, such as HT-29, Saos2, MCF7, HeLa, A549, and more, reflecting the potential of these nano-formulations in cancer therapy. In vivo studies showcased the effectiveness of nano-delivered herbal compounds on tumor-bearing animal models. These experiments included assessing antitumor effects, biodistribution, and pharmacokinetics. Importantly, repeated cell lines such as 4T1 and MCF-7 were used in in vivo studies, indicating the translational potential of these nanoformulations in preclinical research. The overall results demonstrated the significant potential of nano transportation strategies for herbal conjugation in enhancing their bioavailability and therapeutic efficacy. These systems exhibited cytotoxic impressions on different cancerous cells, showing their ability as anticancer agents. Moreover, the in vivo evaluation supported the translational value of these formulations, showing promising antitumor effects and favorable biodistribution. In the context of treatment for cancer along with various disorders, our results highlight the relevance of nano delivery systems in using the curative abilities of herbal medicines.

### Advanced nanocarrier strategies for enhanced herbal anticancer therapy

#### Advanced targeting mechanisms for herbal anticancer delivery

The therapeutic potential of anticancer herbal bioactives is often limited by their inherent pharmacokinetic limitations and nonspecific distribution; however, these challenges can be addressed by engineering advanced nanocarriers with sophisticated "smart" functionalities for targeted, spatiotemporally controlled drug release [119]. These approaches are broadly categorized as passive and active targeting, which are frequently combined with stimuli-responsive systems for precision therapy. Passive targeting exploits the distinctive pathophysiology of solid tumors, notably the Enhanced Permeability and Retention (EPR) effect, wherein the disorganized, hyperpermeable vasculature (with fenestrations ranging from 100 to 2 μm) and compromised lymphatic drainage allow nanocarriers of an optimal size (10–200 nm) to extravasate and accumulate selectively within the tumor interstitium [213]. Surface properties are crucial, as coatings like polyethylene glycol (PEG) confer a stealth characteristic by minimizing opsonization and recognition by the mononuclear phagocytic system (MPS), thereby prolonging circulation time and enhancing tumor accumulation via the EPR effect [214]. To further improve specificity and cellular uptake, active targeting strategies functionalize nanocarrier surfaces with ligands that bind to receptors overexpressed on cancer cells, such as folic acid for the folate receptor (FR-α) [215] or transferrin for the transferrin receptor (TfR1) [216], promoting receptor-mediated endocytosis. This was exemplified by the significantly enhanced cytotoxicity and cellular uptake achieved through the co-delivery of genistein and doxorubicin via targeted lipo-polymeric nanoconstructs in MDA-MB-231 cells [217, 218].

#### Tumor microenvironment-responsive (smart) carriers

The tumor microenvironment (TME) exhibits biochemical properties distinct from those of healthy tissues, a feature exploited by smart nanocarriers designed to remain inert in circulation yet undergo triggered structural changes or degradation for precise drug release at the tumor site [219–221]. One key strategy leverages the acidic extracellular pH of tumors (~ 6.5–6.8), a consequence of aerobic glycolysis (the Warburg effect), and the even lower pH of endo/lysosomal compartments (~ 5.0–6.0). pH-responsive systems incorporate ionizable groups or acid-labile bonds that destabilize under these conditions; for instance, pH-sensitive liposomes composed of lipids like DOPE transition from a lamellar to a hexagonal phase at low pH, facilitating content release [222]. Conversely, redox-responsive carriers capitalize on the dramatically elevated intracellular concentrations of glutathione (GSH) in cancer cells. These systems are engineered with disulfide (–S–S–) bonds that remain stable extracellularly but are cleaved by the high cytosolic GSH levels, resulting in carrier disintegration and cytoplasmic drug release [223]. Furthermore, enzyme-responsive nanocarriers are designed with substrates for tumor-overexpressed enzymes such as matrix metalloproteinases (MMPs) or hyaluronidases. Enzymatic cleavage of these substrates, for example the degradation of a hyaluronic acid-based shell by hyaluronidase, compromises the carrier's integrity and triggers drug release, often while simultaneously enabling receptor targeting [224]. Lastly, to address the severely hypoxic regions common in solid tumors, hypoxia-responsive systems incorporate groups like nitroimidazole or azobenzene, which are reduced under low oxygen tension, becoming hydrophilic and causing carrier breakdown for targeted drug release in these resistant areas [225, 226].

#### The necessity of nanocarriers for herbal anticancer applications

The transition to advanced nanocarrier systems represents a crucial step for the successful clinical translation of herbal bioactives in oncology, as their inherent limitations—including poor aqueous solubility, chemical instability, rapid clearance, and non-specific toxicity—severely diminish their efficacy as free agents. Nanocarriers directly counter these challenges through several mechanisms: they enhance solubilization and stability by encapsulating hydrophobic compounds like curcumin or artemisinin within liposomes or polymeric nanoparticles, thereby protecting them from degradation [227–229]; they improve bioavailability and extend circulation times via nano-sizing and surface modifications like PEGylation, which reduce renal filtration and mononuclear phagocyte system uptake, promoting tumor accumulation through the enhanced permeability and retention (EPR) effect; and they enable targeted delivery and reduced toxicity by utilizing ligands for active targeting and stimuli-responsive release within the tumor microenvironment (TME), which concentrates the drug at the disease site while sparing healthy tissues. Furthermore, nanocarriers can overcome multidrug resistance (MDR) by evading efflux pumps such as P-glycoprotein and allow for the co-delivery of chemotherapeutic agents with chemo-sensitizing herbal compounds like ginsenoside Rg3 for synergistic therapy [230]. Consequently, the progression from simple encapsulation to intelligently engineered, multifunctional nanocarriers is fundamental to realizing the full potential of herbal medicines, transforming them into viable, effective, and safe anticancer therapeutics [231].

## Wound healing applications

Preparations from traditional medicinal plants have long been utilized for wound healing purposes, addressing a diverse array of skin-related diseases. Herbal treatments in wound healing encompass a multifaceted approach involving disinfection, debridement, and the provision of a conducive condition to assist the natural healing process [1].

Recent research endeavours have increasingly focused on investigating the wound healing effects of herbal substances in combination with various nanocarriers. Nanocarriers provide an auspicious path for improving the delivery and efficacy of herbal agents, thereby augmenting their therapeutic potential in wound healing applications.

This section focuses on the emerging field of study that investigates the combined impact of herbal components and nanocarriers on wound healing. We analyze widely used herbal substances and nanocarriers, presenting specific instances from recent research that showcase their ability to enhance wound healing mechanisms.

### Wound healing herbal compounds

#### Aloe vera

Aloe vera, a succulent plant with a history as a natural remedy, has remarkable wound-healing properties. Its gel-like interior contains acemannan, a polysaccharide that stimulates tissue regeneration. Aloe vera enhances the production of growth factors (GFs) and collagen, the essential components for tissue repair. Acemannan activates the cellular machinery to increase collagen synthesis and promote new tissue growth. This helps the wound contract and close, a critical step in healing. This herbal remedy modulates inflammation, a natural but sometimes harmful response to injury. Aloe vera has anti-inflammatory characteristics that reduce the inflammatory response, creating a better environment for healing. It also protects the wound from oxidative stress and microbial infections, which can impede healing or cause complications. Aloe vera has antioxidant, antibacterial, and antifungal properties that shield the wound from these threats [232].

#### Calendula

**Fig. 5:**
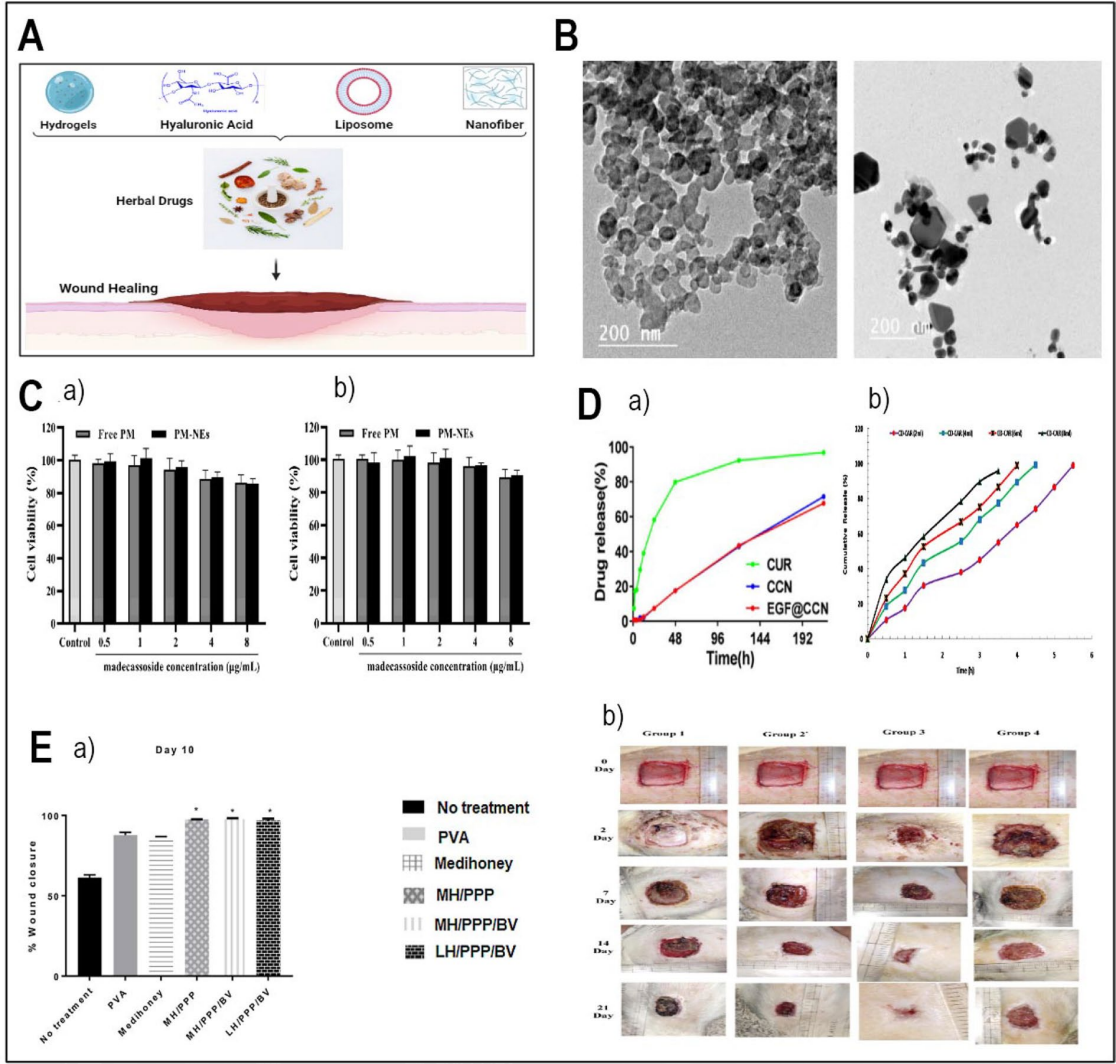
**A** The mechanism of delivering herbal drugs to wounds with different carriers. **B** TEM image of chitosan nanoparticles and bioactive Alo vera chitosan nanoparticles poly load (Redesigned from reference [252] with permission). **C **(**a**) In vitro cytotoxicity of PM-NEs against HaCaT cells and (**b**) RAW264.7 cells [242]. **D **(**a**) cytotoxicity assay of CS-NPS and EGF@CS-NPS on human fibroblasts (Redesigned from reference [248] with permission). (**b**) Comparative in-vitro drug release study of all hydrogels loaded with different concentration of propolis (Redesigned from reference [55] with permission). **E **(**a**) Bar chart showing wound closure in different groups. almost 98% reduction in wound size by day 10 compared to PVA and no treatment controls (Redesigned from reference [244] with permission). (**b**) The macroscopic alterations of the wound were observed over various post-excision days in Group I (comprising non-treated animals), Group II (involving collagen film without treatment of CFE), Group III (undergoing treatment with CFE-loaded collagen film), and Group IV (receiving treatment with a commercial product) (Redesigned from reference [236] with permission). (p-value = p, *p < 0.05, **p < 0.01, *** p < 0.001)

Calendula (CAD), a flowering plant with a history in wound healing, has remarkable properties. Its natural compounds, such as flavonoids, triterpenoids, and carotenoids [233, 234], modulate healing processes. Calendula balances inflammation, an essential but sometimes harmful response to injury. It reduces inflammation, creating a better environment for healing. It also accelerates wound healing by stimulating angiogenesis—the formation of new blood vessels. This provides the wound with nourishment and oxygen, speeding up tissue repair and regeneration [235]. Moreover, calendula enhances the formation of granulation tissue, a scaffold for new tissue growth. It protects the wound from infections and oxidative stress, which can impede healing. It has antimicrobial and antioxidant properties that shield the wound from these threats. In the modern era, calendula has been used in advanced delivery systems, such as nanoformulations. A research conducted by Rathod and colleagues in 2022 involved the preparation of a calendula flower extract loaded collagen film for the purpose of serving as a safe and efficient wound dressing. The collagen film fabrication process utilized the solvent casting method and underwent thorough characterization to evaluate various physicochemical properties such as water absorption capacity, total phenolic content, total flavonoid content, antioxidant activity, among others. Figure 5E part b displays images depicting the wound contraction area at various time points post-excision. Findings suggested that both the placebo and control groups exhibited lower re-epithelization rates compared to both the formulation group and the group utilizing a commercially available sample. These techniques improve the bioavailability and delivery of calendula’s healing compounds. Calendula remains a natural healer, offering faster recovery and healthier skin [236].

#### Gotu kola

Gotu kola, an herb with a legacy in traditional medicine, plays a part in wound healing and skin rejuvenation. Its main compound, asiaticoside, stimulates regenerative processes [237]. It enhances wound healing by activating fibroblasts, the cells that produce collagen, the structural protein of the skin. Asiaticoside increases fibroblast proliferation and collagen synthesis, improving wound strength and resilience (Table 3) [238].

**Table 3:** Summary of studies in the existing literature utilizing nano-delivery systems for herbal compounds for wound-healing application

Name of the compound	Bioactive compound	Nanodelivery method	Fabrication of the carrier	In vitro and in vivo assays	Cell lines used	Refs.
Aloe vera	Aloe vera	AV-insulin gel	Nanoemulsion formulation selection	In vitro and Ex vivo and in vivo assays	Adult albino Wistar rats	[250]
Aloe vera	Aloe vera	CS-AV gel		MIC for antibacterial studies, Hemocompatibility evaluation, MTT assay, In vivo wound healing study	3T3 murine fibroblast cells, male Wistar rats	[251]
Aloe vera	Aloe vera	CS-based NPs	Ionic gelation method	Antibacterial activity using colony, MTT assay	–	[252]
Calendula	Carotenoids	Carrot and marigold extract nanostructured lipid carriers	Melt-emulsification coupled with high pressure homogenization	In vitro, MTT, LDH assay and Optical microscopy, etc.	L929 cells	[253]
Calendula	Calendula and coffee extract	Co-delivery of CAD and coffee extracts by PVA/PCL	Electrospinning method	In vitro, Antioxidant assay via DPPH radical scavenging assay, In vitro cytotoxicity analysis via Alamar Blue assay, In vitro wound healing assay	Human dermal *fibroblast*	[254]
	Calendula officinalis flower extract	CS-sodium alginate- PVA hydrogel		In-vitro, agar diffusion technique	*Acinetobacter baumannii*, Staphylococcus epidermidis, Proteus mirabilis	[255]
	Calendula	CAD-CS-AV hydrogel	Hydrogel crosslinking and nano-liposomes of soy lecithin added to the hydrogel	In vitro characterization, MTT	Fibroblast cells (L929)	[256]
	Calendula	Collagen film	Solvent casting method	Hydrogel properties, in-vitro cell experiments Antioxidant efficacy, water content, MTT, *In-vivo* wound healing	Swiss 3T3 albino fibroblasts, Male Sprague–Dawley (SD) rats	[236]
Gotu kola	Asiaticoside	PVA-CS	Electrospinning technique	In vitro and in vivo assays		[238]
Gotu kola	Madecassoside	Paeonol and Madecassoside with noemulsions	High-pressure homogenization technique	In vitro cytotoxicity, Cellular Proliferation and Migration Assay, Cellular Barrier Repair Study, Clinical Trial, etc	HaCaT, RAW264.7	[242]
Honey	Honey	PVA, egg white, montmorillonite (MMT) nanoclay	Suspension method	In vitro release studies and cytotoxicity by flow cytometry, in vivo assays, Tensile Test, etc	Human peripheral blood mononuclear cells (PBMC), BALB/c mice	[257]
	Honey beepropolis extract	Polymer-based Hydrogel		In vitro antibacterial activity	Gram-positive bacteria (*S. aureus*) and Gram-negative bacteria (*Pseudomonas aeruginosa*) and (fungus) *Aspergillus flavus* and *Candida albicans*	[55]
	Manuka honey	PVA, CMC, gelatin, and starch	Casting method	In vitro characterisation of activity	*Staphylococcus aureus*	[258]
Honey	Honey	Hydrogel with Carbopol-940 and Chitosan	Cold mechanical method	In vitro characterisation of Cytotoxicity studies via MTT and i*n-vivo* wound healing	L929 cell lines (mouse fibroblasts), Albino Wistar rats	[259]
Honey	Honey	Co-delivery of honey and Nepeta dschuparensis plant with polyvinyl alcohol and chitosan (PVA/Chit) nanofiber mats	Electrospinning method	In vitro and in vivo characterisation	Male Wistar rats	[260]
Honey	Honey	Honey-curcumin-PVA multilayered polyvinyl alcohol/cellulose acetate	Electrospinning method	In vitro characterisation of activity via agar disc diffusion	*Escherichia coli*	[261]
	Honey	Alginate/PVA-based nanofibers	Electrospinning method	In vitro characterisation of the formulation and activity study via agar disc diffusion, Antioxidant study, and MTT	NIH/3T3 cells	[262]
	Honey	PVA-based	Electrospinning method	In vitro characterisation of activity study via Viable cell count method, MTT, In vivo wound healing assay	L929 mouse fibroblast cells (ATCC), female Sprague–Dawley rats (*Rattus norvegicus albinus*)	[244]
	Honey	Curcuma longa-honey with mupirocin-loaded niosomal gel	Ether injectionmethod	In vitro and in-vivo wound healing study	Wistar albino rats	[263]
Turmeric	Curcumin	Liposomes	Thin film hydration method	In vitro and in vivo skin penetration study	Albino Wistar rats	[264]
	Curcumin	CS-PVA-carbopol-PCL	Dual electrospinning method	MTT analysis and morphological studies	Buccal fat pad-derived mesenchymal stem cells	[265]
	Curcumin	Curcumin-cerium oxide hydrogel		Haemolysis assay, MTT, Live and dead staining, Antioxidant studies, ROS analysis, and In-vivo anti-inflammatory test, etc	NIH 3T3 cells (Normal murine fibroblast) and HaCat cells (human keratinocyte cell line)	[266]
		CS-PVA-PCL	Electrospinning method	In vitro characterisation of the formulation and morphological, MTT, in vivo studies of the wound healing	Buccal fat pad-derived mesenchymal stem cells	[267]
	Curcumin	Co-delivery of curcumin and resveratrol with nanoemulsion based gel system (nanoemulgel)	The spontaneous emulsification method	In vitro, ex*-vivo* skin deposition, and *in-vivo* burn healing potential tested	Adult male Wister rats, male albino rat	[268]
		Curcumin-CS-EGF spray	The ion crosslinking method	In vitro characterization, MTT, in vivo wound healing	Human fibroblast cell lines, Wistar rats, Sprague–Dawley males	[248]

Gotu Kola also modulates inflammation and oxidative stress, which can delay healing. It reduces inflammatory factors like PGE2, minimizing swelling, redness, and discomfort [239]. Gotu Kola can treat keloids, scars that grow beyond the wound margins due to excessive collagen. It regulates collagen synthesis and angiogenesis, the formation of new blood vessels. Angiogenesis provides the wound with nourishment and oxygen, essential for repair [240, 241]. several nanoformulations have been employed for the delivery of gotu cola. In a recent investigation undertaken by Lu et al. in 2023, nanoemulsions (PM-NEs) were formulated containing paeonol and madecassoside to enhance their delivery effectiveness, as well as to facilitate sensitive skin repair and anti-inflammatory effects. The results depicted in Fig. 5C part a,b reveal that when immortalized human keratinocytes (HaCaT) and mouse mononuclear macrophage (RAW264.7) cells were exposed to free PM or PM-NEs with madecassoside concentrations ranging from 0.5 to 8 μg/mL for 24 h, the cell viabilities were consistently maintained above 80%, indicating minimal observed cytotoxicity. Furthermore, the PM-NEs were successful in improving the transdermal penetration and retention of paeonol and madecassoside glucoside, while also improving cellular uptake [242].

Gotu kola’s potential can be optimised by novel delivery systems, such as nano formulations. These carriers enhance and target its therapeutic effects, offering new possibilities in wound healing and skincare.

#### Honey

Honey is a natural substance that has been used for wound healing for centuries. Honey has many properties that can benefit the wound healing process, such as antimicrobial, anti-inflammatory, antioxidant, and osmotic effects. Honey can retard the growth of bacteria and fungi that can infect the wound, reduce the swelling and pain caused by inflammation, protect the wound from oxidative damage, and stimulate the removal of dead tissue and fluids from the wound [243]. However, honey also possesses certain drawbacks, including low solubility, poor stability, and variable composition. To overcome these challenges, nanoformulations of honey have been developed to enhance its delivery and efficacy. Nanoformulations are tiny particles that can encapsulate honey or its active compounds and deliver them to the wound site more effectively and precisely. in a study undertaken by Sharaf et al., (2019), a delivery system was developed based on a blend of biodegradable polymeric hydrogel, specifically κ-carrageenan (κ-CAR) and β-cyclodextrin (β-CD), to encapsulate HBP. The synthesized hydrogels underwent thorough characterization to investigate both their surface and internal morphology. Figure 5D part b illustrated the in-vitro release profile of HBP from κ–CAR/β-CD hydrogels. The cationized cotton fabric treated with κ–CAR/β-CD/HBP showed promising prospects in the field of wound healing textiles [55]. another research conducted by Zekry et al., (2020) utilized Honey, pomegranate peel extract, and bee venom in conjunction with polyvinyl alcohol for the creation of a pioneering nanofibrous wound dressing. The effectiveness of the formulations was determined by their antibacterial properties, cytotoxic effects, and wound healing potential using an excisional wound rat model. The results indicated that all three therapeutic scaffolds led to a significant increase in wound closure percentage compared to both the PVA-treated group and the untreated group by day 10, as illustrated in Fig. 5E part a [244]. Nanoformulations can also enhance the stability and protection of honey against degradation, control its release pattern, and reduce its immunogenicity and toxicity [245].

#### Turmeric

Turmeric, a spice with culinary and medicinal uses, plays a part in wound healing and skin rejuvenation. Its main compound, curcumin, is a polyphenol that modulates healing processes [6, 235, 246]. Curcumin inhibits bacterial growth by disrupting their membranes and metabolism, protecting the wound from infection. It also reduces inflammation and oxidative stress, which can delay healing. Curcumin enhances wound healing by regulating molecules, curbing inflammation, and increasing collagen deposition [247]. However, curcumin has poor solubility and bioavailability, limiting its application. To overcome this, novel products such as microemulsions, liposomes, micelles, nanoparticles, and nano lipid carriers have been developed to improve curcumin delivery systems. These products promise longer circulation, enhanced permeability, and increased resistance to degradation, addressing the issue of curcumin bioavailability. a study conducted by Li and colleagues in 2021 Unveiled a pioneering nano-drug delivery system tailored for addressing skin imperfections via spray application. The researchers formulated chitosan nanoparticles loaded with curcumin and modified them with epidermal growth factor (EGF) to create an aqueous EGF-CUR-loaded chitosan nanoparticles (EGF@CC NPs) specifically for addressing dermal injuries. The release profiles of the drugs free-CUR, CC, and EGF@CC NPs are depicted in Fig. 5D part a. The results demonstrate that on the first day, free-CUR exhibited a release of 58%, while only 7% was released from CC NPs and EGF@CC NPs [248]. These curcumin formulations, using nanotechnology delivery systems, may amplify the wound-healing potential of turmeric [249].

### Delivery of herbal compounds in wound healing applications

Effective wound healing is essential for preventing infections and promoting the recovery of injured tissues. While traditional wound dressings have been used for years, they often fall short in providing ideal conditions for healing, especially for severe or chronic wounds. Recent advancements in wound dressing materials aim to address these shortcomings and accelerate the wound healing process while simultaneously preventing bacterial infections [269]. This section explores various nanocarriers and delivery systems used for the targeted delivery of herbal compounds in wound healing applications, with a focus on their potential benefits.

#### Membrane nanocomposite for wound healing

To optimize the effectiveness of wound healing, carriers that can deliver herbal compounds such as asiaticoside with controlled release profiles are essential. Membrane nanocomposites, designed to mimic the body's extracellular tissue, have surfaced as a promising drug delivery form. These nano-composites are tailored to enhance the wound healing process and have been shown to be particularly effective in delivering herbal compounds [238].

#### Chitosan nanoparticles

Chitosan nanoparticles are used in various drug delivery applications. In a 2022 study by Salama et al., the objective was to create a nano carrier for Aloe vera extract using CS NPs. The CS NPs were carefully prepared and then loaded with Aloe vera extract to form Aloe vera-laden chitosan nanoparticle nanocomposites. These nanocomposites exhibit excellent antibacterial properties while minimizing toxicity. TEM images of blank and aloe vera-loaded NPs are shown in Fig. 5B. In conclusion the identified formulation could be regarded as a crucial tool in combating medication resistance in cancer [252].

#### Hydrogels

Hydrogels have attracted considerable interest in wound healing research owing to their distinctive characteristics and versatility. Polymeric hydrogels have found extensive application in biomedicine and tissue engineering. These hydrogels possess ECM-mimicking properties such as high water retention (up to thousands of times their weight), biocompatibility, biodegradability, and the ability to support cell growth during tissue regeneration [270, 271]. Moreover, Recently, 3D-printed hydrogels have demonstrated significant efficacy in promoting wound healing, showcasing their promising potential in advanced biomedical applications [272].

#### Liposomes

Curcumin, a potent wound healing agent, is often limited by its low bioavailability when administered orally. Therefore, topical application is preferred for effective wound treatment. Liposomes, lipid-based nanocarriers, have demonstrated efficacy in delivering curcumin for burn therapy. By encapsulating curcumin within liposomes, the bioavailability and therapeutic potential of this herbal compound can be maximized for wound healing applications [270].

#### Nanofibers

Nanofibers, characterized by their small diameter (approximately 10–100 nm) and large specific surface area, offer unique qualities for wound healing applications. These nanofibers are produced through electrospinning, a process that uses electric force to generate nanoscale fibers from various polymer solutions. Nanofibers have been explored as promising wound dressings and carriers for herbal compounds due to their ability to create a scaffold-like structure that supports tissue regeneration. The high surface area of nanofibers can enhance the delivery and release of herbal compounds, contributing to improved wound healing outcomes [270, 273].

### In vitro and in vivo results

The nano delivery of herbal compounds for wound healing applications has exhibited promising outcomes in various in vitro and in vivo studies summarised in Table 3. Aloe vera, in different formulations such as a topical gel with insulin-loaded nanoemulsion and chitosan hydrogel containing Aloe vera gel and EDTA, demonstrated significant wound healing potential in diabetic rats and murine fibroblast cells, respectively. Carotenoids delivered through nanostructured lipid carriers showed notable antioxidant and anti-inflammatory actions on L929 cells. Calendula, whether in the form of nanofibrous mats, collagen film, or hydrogel with Ag_2_O/SiO_2_, exhibited antibacterial efficacy against Acinetobacter baumannii and Staphylococcus epidermidis. Asiaticoside, delivered through a membrane nanocomposite, demonstrated effective wound healing in in vivo rabbit models. Madecassoside, delivered in nanoemulsions, showed promising results in in vitro studies, including cellular proliferation and migration assays, as well as three-dimensional skin model efficacy studies. Various forms of honey, such as those incorporated into PVA-based nanocomposites, carrageenan/β-cyclodextrin hydrogels, and PVA/sodium carboxymethylcellulose/gelatin/starch-based gels, displayed in vitro and in vivo wound healing activities. Curcumin-loaded liposomes and nanofibrous scaffolds, as well as co-delivery with cerium oxide in a nano-hybrid hydrogel system, demonstrated enhanced wound healing effects in Albino Wistar rats. Curcumin-loaded chitosan nanoparticles modified with EGF spray exhibited notable antibacterial activity against *E. coli* and *S. aureus*, along with accelerated wound healing in Wistar rats.

These studies collectively suggest that nano delivery systems improve the therapeutic effectiveness of herbal substances in wound healing applications. The most frequently used method across these studies involved in vitro characterizations of the formulations, including cytotoxicity assessments and antioxidant assays. In vivo assays were commonly conducted on animal models such as Wistar rats, Sprague–Dawley rats, and rabbits, with repeated cell lines including L929 cells, fibroblast cells, and human dermal fibroblasts. Overall, The findings emphasize the potential of nano delivery systems in improving the wound healing properties of herbal compounds, showcasing antibacterial, antioxidant, and anti-inflammatory effects in both in vitro and in vivo settings.

### Analysis of herbal compounds in wound healing: fundamentals and therapeutic mechanisms

The process of wound healing is a highly orchestrated sequence of overlapping phases—haemostasis, inflammation, proliferation, and remodelling—driven by a complex interplay of cells and critical signalling molecules such as growth factors [274]. Vascular Endothelial Growth Factor (VEGF) is the primary mediator of angiogenesis, while Transforming Growth Factor-Beta (TGF-β) promotes fibroblast migration, proliferation, and extracellular matrix (ECM) synthesis [275–277]. Nanocarriers significantly enhance the therapeutic potential of herbal compounds by ensuring their targeted and sustained delivery to the wound site while actively modulating this biochemical signalling landscape. For instance, nano-encapsulated curcumin upregulates VEGF and TGF-β1 expression, enhancing angiogenesis and granulation tissue formation [278–280]. Similarly, Aloe vera nano-formulations sustain the release of bioactive compounds, increasing VEGF and FGF-2 expression to accelerate re-epithelialization [281–284]. Calendula-integrated nanofibrous scaffolds promote fibroblast migration and upregulate TGF-β secretion for improved ECM synthesis [285], whereas Honey bee propolis extract (HBP) in a biodegradable hydrogel exhibits broad antibacterial activity and sustained flavonoid release, which modulates inflammation and promotes healing by stimulating fibroblast production of VEGF and TGF-β, supporting the shift from inflammation to tissue proliferation [286]. Also, nanoemulsions of asiaticoside potentiate the Smad signalling pathway downstream of TGF-β receptors, boosting collagen synthesis and tensile strength [287, 288]. Moreover, quercetin-loaded lipid nanoparticles enhance wound healing by improving drug bioavailability, promoting sustained release, and supporting skin cell viability and migration. These nanoparticles facilitate controlled quercetin delivery, reduce toxicity, and increase skin permeation, thereby accelerating tissue repair and regeneration [289].Thus, by overcoming the inherent bioavailability limitations of phytochemicals and precisely influencing growth factor dynamics, nanocarriers promote a more efficient and complete healing process.

## Tissue engineering applications

The field of tissue engineering is complex and involves experts from several fields such as materials engineering, mechanical engineering, medicine, and other branches of biosciences [290]. Various scaffolds made from different materials have been used in tissue engineering [291]. Regardless of the type of tissue, creating a scaffold requires careful consideration of numerous important factors. Among these characteristics are mechanical qualities, biocompatibility, and the synthesis technique applied [291, 292]. Usually, scaffolds studies make use of classifications of drugs, such polymers and ceramics.

**Fig. 6:**
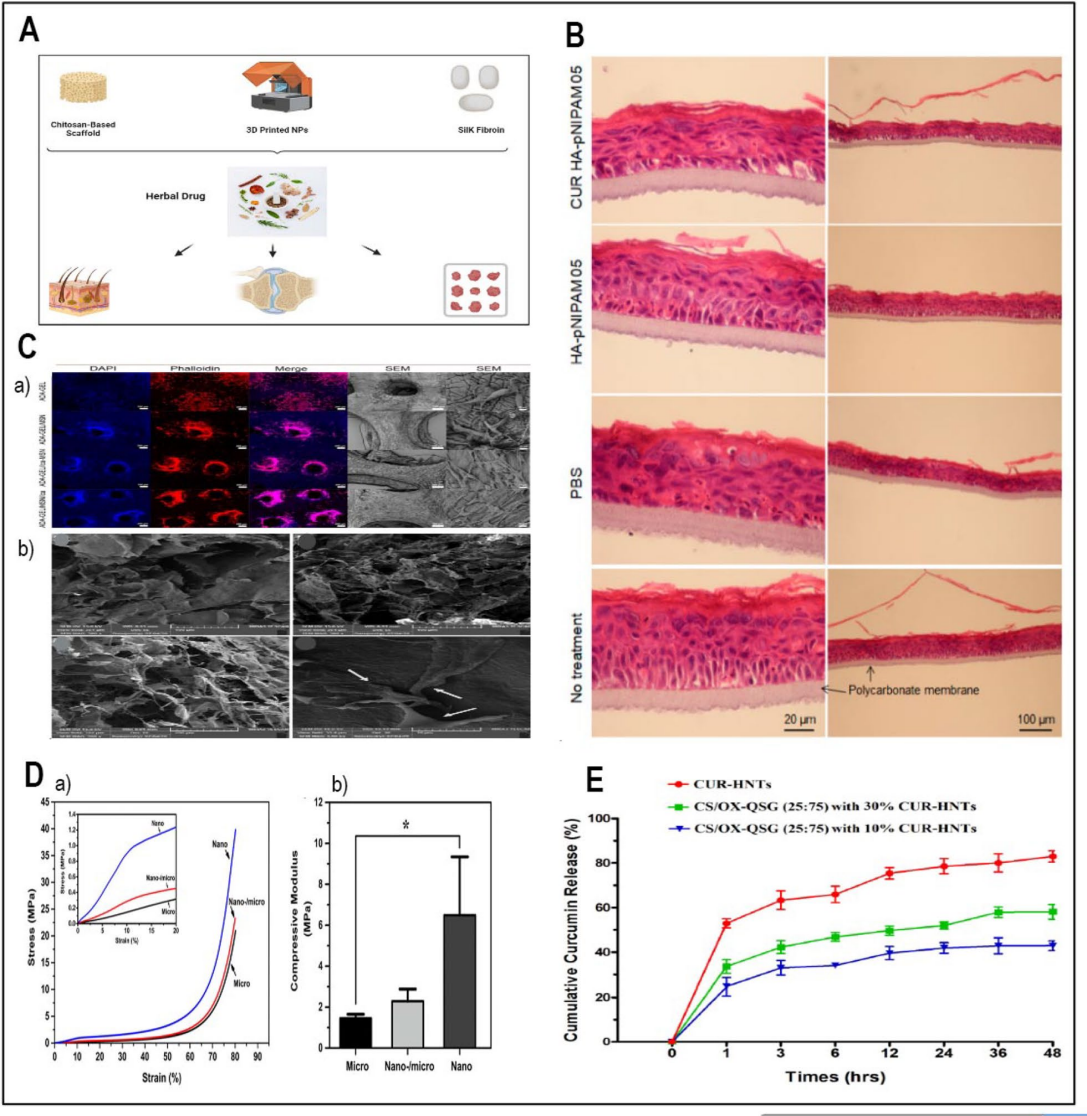
**A** The application of herbal compounds in the field of tissue engineering (Designed by BioRender). **B** Histological examination to evaluate acute skin irritation. The EpiSkin® sample was stained using Hematoxylin and Eosin (H&E). The scale bars for the left and right columns are labeled as 20 μm and 100 μm, respectively (Redesigned from reference [316] with permission). **C** The morphology of the adherent MC3T3-E1 cells was visualized using fluorescence microscopy with Phalloidin/DAPI staining. The scale bars in the images represent 200 μm. Additionally, SEM was performed after 4 days of incubation to further examine the cells. The scale bars in the SEM images are 100 μm (left column) and 10 μm (right column) (**a**) (Redesigned from reference [309] with permission). SEM photos of hydrogels made from Chitosan/Oxidized quince seed gum (CS/OX-QSG) and NIH3T3 fibroblast cell attachment on optimal Curcumin-HNTs@hydrogel (**b**) (Redesigned from reference [297] with permission). **D** Compressive characteristics of rolled scaffolds made from nano-/microfibrous, microfibrous, and nanofibrous materials. Representative compressive stress–strain plot (**a**), compressive modulus of 3-D scaffolds. (**b**) (Redesigned from reference [312] with permission). **E** The total amount of CUR released from HNTs and the optimal hydrogel (Chitosan/Oxidized quince seed gum CS/OX-QSG with a ratio of 25:75) including 10% and 30% CUR-HNTs (Redesigned from reference [297] with permission). (p-value = p, *p < 0.05, **p < 0.01, *** p < 0.001)

Plant-based materials, such as natural biopolymers, play an important part in the development of cellular activity, especially in relation to chemical signals and compatibility with biological systems [293, 294]. Recently, the combination of TE and herbal therapy has experienced significant expansion. Consequences on tissue engineering of herbal substances combined with different nanocarriers have been attracting more and more interest from researchers. Researchers want to develop better therapeutic approaches for tissue regeneration by integrating the specific features of nanocarriers, such focused distribution and controlled release, with the bioactive elements of herbal medicines. This section mostly focuses on the growing area of study on the combined effects of herbal substances and nanocarriers in tissue engineering. We review often used herbal compounds and nanocarriers, stressing specific cases from recent studies demonstrating their ability to enhance tissue repair and regeneration processes. Figure 6A shows some materials employed in tissue engineering as herbal medication vehicle.

### Tissue engineering herbal compounds

#### Curcumin

The amazing polyphenol derived from the golden spice turmeric, curcumin, has far more uses in the field of tissue engineering than only in cooking. In the field of regenerative medicine, curcumin reveals its promise in addition to its well-known anti-inflammatory, antioxidant, and anti-tumoractivities. More especially, curcumin's ability to support cartilage tissue engineering has attracted a lot of interest [295]. As Ahangari et al. (2019) clarify, curcumin has moved outside its conventional focus on wound healing and into a more general role in the regeneration of many tissues, including tendons and bones. A convincing approach for tissue engineering uses is the coupling of curcumin with appropriate biomaterial scaffolds, like polymeric matrix [247]. This synergy promises to be able to use curcumin's many properties to induce tissue regeneration. Curcumin's tissue engineering potential is especially notable in the field of bone healing. Verma et al. (2019) examined curcumin's effectiveness against cancer cells while showing low toxicity towards normal cells (Table 4) [296]. Using extremely comparable to the bone substitutes, carbonated apatite (CA) NPs, curcumin finds an ally in improving bone healing. The great anti-inflammatory qualities of curcumin, as shown by the BSA protein denaturation test, along with its anti-cancer action against human osteosarcoma cells, expose its tissue engineering ability, promising not only to repair but also restoration. Moreover, curcumin enters interesting partnerships as demonstrated by its co-delivery with Quince (Cydonia oblonga) [297] (Table 4). Rich in gum, the Quince seeds produce quince seed gum (QSG), full in galacturonic and glucuronic acids. This wealth of functional groups offers several sites for monomer crosslinks and reactions. As Fig. 6C (b) shows, the hydrogels displayed a porous structure.

**Table 4:** Summary of studies in the existing literature utilizing nano-delivery systems for herbal compounds for tissue engineering application

Name of the compound	Bioactive compound	Nanodelivery method	Fabrication of the carrier	In vitro and in vivo assays	Cell lines used	Refs.
Turmeric	Curcumin	Carbonated apatite nanocarriers		In vitro characterisation of the formulation, protein denaturation assay and cytotoxicity assay	MG-63 human osteosarcoma cells	[296]
	Curcumin	Curcumin- quince seed gum codelivery via CS-based NPs hydrogels		In vitro characterisation of the formulation, cytotoxicity assay by MTT against NIH-3 T3 cells, Cell adhesion study, Antibacterial test via disk diffusion method	NIH3T3 fibroblast cells	[297]
	Curcumin	ZSM-5 nanozeolite -PCL/Gelatin nanofibers	Electrospinning method	In vitro characterization, cytotoxicity assay (MTT), proliferation quantitation (PicoGreen), cell adhesion study, qPCR, etc	Human adipose-derived stem cells	[305]
	Curcumin	Niosome	Thin-film hydration technique	In vitro characterisation of the formulation, behavioral assessments	NS/PCs were isolated from the human fetal brain, male Wistar rats	[298]
Icariin	Icariin	Fibrin- ICA –PLGA		In vitro characterisation of the formulation, cytotoxicity assay by MTT against hADSCs, gene analysis	hADSCs	[306]
	Icariin		Emulsion template method	In vitro characterization, protein absorption, cytotoxicity, proliferation analysis, phenotype detection, etc	Osteoblasts cultured from SD male rats	[307]
	Icariin	CS- nanotubes hydrogel	Sol–gel transition	In vitro characterisation of the formulation, cytotoxicity assay by MTT, In vitro osteogenic differentiation analysis	hASCs	[308]
	Icariin	Alginate dialdehyde-gelatin with silica-calcia NPs	3D sol–gel printing with Alginate-gelatin (with/without MSNs)	In vitro characterisation of the formulation, live-dead assay, and osteogenic differentiation, Mineralization assay, etc	Preosteoblastic cell line MC3T3-E1	[309]
	Icariin	ICA-moxifloxacin hydrochloride-PCL	Coaxial electrospinning method	In vitro characterisation of the formulation, In vitro antibacterial evaluation, MTT, osteogenic differentiation, In vivo osteogenesis evaluation	MC3T3-E1 cells	[310]
	Icariin	ICA-HA based NPs	Coaxial electrospinning method	In vitro characterisation, MTT staining and calcium deposition test, etc	Human fetal osteoblasts (HFOBs)	[311]
Gensing	Ginsenoside Rg1	SF-PCL and PCL	Double-electrospinning method	In vitro characterisation, Morphological study, Compressive stress–strain measurements, MTT, etc	Mouse osteoblast-like MC3T3-E1	[312]
Resveratrol	Resveratrol	PCL based	Electrospinning method	Echocardiography, Histopathology, Immunofluorescence, and Immunohistochemistr analysis, etc	Adult ICR-CD1 mice	[304]
	Resveratrol	Levan-based	Electrohydrodynamic atomization (EHDA) technique	In vitro Biocompatibility Assay via WST-1, study of gowth and attachment of PCS-201–012	Human dermal fibroblast cell lines (PCS-201–012)	[313]
	Resveratrol	Levan-based	HL hydrolysis via microwave-assisted acid hydrolysis	In vitro Biocompatibility Assay via WST-1, study of attachment of HaCaT cells	HaCaT cells	[314]

Hydrogels with a high concentration of OX-QSG exhibited a compact and porous framework having fewer openings due to the increased density of cross-linking caused by the aldehyde-functionalized QSG. The NIH3T3 fibroblast cells adhered effectively to the hydrogel surface, indicating a strong degree of cellular affinity for hydrogels adhesion [297]. The release profiles displayed over a period of 48 h can be observed in Fig. 6E. The release of CUR from hydrogels occurs through the decomposition of the polymer matrix, resulting in its release. Thus, CUR- halloysite nanotubes (HNTs) have a relatively high CUR delivery, which have discharged around 80% within a 48-h period. The ideal formulation exhibited a comparatively gradual release of CUR over a period of 48 h. It is evident that increasing the level of CUR-HNTs (30%) added to the carrier leads to a higher amount of released medication. The discharge rates of carriers with 30% and 10% CUR-HNTs were determined to be approximately 57% and 40%, respectively [297]. The hydrolysis of QSG demonstrates its potential as a beneficial component in tissue engineering, therefore driving curcumin towards novel therapeutic vistas [297]. Narouiepour et al., 2022 investigated how niosomal nanoparticles might be used to transfer curcumin to the brain and enhance neural stem cells therapy for traumatic brain injury [298].

#### Icariin

Strong flavonoid icariin (ICA), derived from the herb Epimedium—also known as horny goat weed—showcases its several uses in the field of tissue development. Beyond its conventional use, ICA is a flexible participant promoting advancements in osteogenesis, angiogenesis, and neurogenesis. Acting as a herbal replacement for GFs, ICA arranges the chondrogenesis promotion in stem cells. From upregulating cartilage-specific genes and improving extracellular matrix (ECM) formation to exerting anti-inflammatory effects, this dynamic process comprises many routes. ICA turns out to be a useful player guiding the route toward regenerated chondrogenesis [299].

Furthermore, the variety of icariin's uses reaches the domain of scaffold building. Under the hands of researchers, ICA becomes a fundamental component in building stable and biocompatible frameworks for TE applications [300]. When combined with ICA, these models help a great variety of cell types—including chondrocytes and embryonic stem cells with the potential to develop cardiac cells—to grow and differentiate. Moreover, ICA can be used to increase the synthesis of important components such as collagen and proteoglycans, which are required for bone and cartilage TE [301]. By means of this cooperation between ICA and hydrogel framework or chemical crosslinking, the regeneration capacity is enhanced and a viable path in tissue restoration is presented. Fundamentally, generated from the Epimedium plant, ICA represents a flexible bioactive molecule with broad applications in tissue engineering and cancer fight.

#### Ginsenoside Rg1

Extracted from the famed ginseng plant, ginsenoside Rg1 is a multifarious molecule with several uses including cardioprotection, neuroprotection, and anti-aging. Its importance reaches even into the field of skin tissue engineering, where it is essential for skin TE and repair. In the context of skin tissue engineering, Ginsenoside Rg1 takes front stage as a strong accelerator for the fibroblast mobility and development, the sentinel cells producing essential components of the ECM. This essential role greatly adds to the tensile strength and structural integrity of the skin, hence Ginsenoside Rg1 is a major actor in promoting the rejuvenation of aged or injured skin [302]. Beyond its function in fibroblast activation, Ginsenoside Rg1 is really good in arranging a symphony of molecular events fit for TE and wound healing. This saponin increases the expression of GFs and cytokines, therefore creating a favorable milieu for cellular regeneration and tissue integrity restoration. Key components in the complex dance of cellular reactions necessary for effective wound healing and tissue remodelling are these GFs and cytokines [303].

#### Resveratrol

Found in abundance in natural foods including grapes, red wine, peanuts, and berries, resveratrol is a polyphenol with amazing adaptability for health. Beyond its standing for reducing diabetes, oxidative stress, and inflammation, resveratrol offers therapeutic value to the field of liver tissue engineering [295, 302]. Resveratrol takes front stage inside hepatocytes, the main workers of the liver, by coordinating a reduction in oxidative stress and inflammation. Often the cause of liver disease and a host of other problems, these two destructive enemies include Resveratrol creates a supportive environment for hepatocytes to flourish and perform their vital roles by quieting the storm of oxidative stress and inflammation. Stimulating the nuclear factor pathway is one of the main processes Resveratrol uses to produce effects on the liver. This essential route controls the production of cytoprotective genes and antioxidant enzymes, therefore serving as a sentinel. Resveratrol helps hepatocytes to sustain their key functions in metabolic and detoxifying processes and to strengthen their defences against oxidative onslaught by means of this activation [295, 302].

Especially, Resveratrol's contributions to tissue engineering reach outside the lab and into the field of clinical trials. Particularly in relation to cardiac and skeletal muscle repair, ongoing studies include the trials listed under clinicaltrials.gov (NCT03525, NCT01914) highlight the growing interest in using Resveratrol's potential for TE [304] (Table 4). Furthermore, the usage of resveratrol relates to wound healing since its considerable ability to lower oxidative stress and inflammation makes a difference. Great potential for wound healing comes from resveratrol-loaded nanoparticles, carefully modified to increase solubility and target bacteria. These low-toxic, highly biocompatible, and highly antioxidant nanoparticles thus demonstrate their capacity to establish a condition suited for wound healing. Applied in gel form, Resveratrol-loaded nanoparticles speed up tissue regeneration, collagen synthesis, and skin cell proliferation, therefore offering a full treatment for complex wounds [302].

### Delivery of herbal compounds in tissue engineering application

The goal of TE is to build frameworks with the capacity to mend, support, or enhance damaged tissues and organs. One essential part of TE is the formation of suitable scaffolds that can imitate the ECM of native tissues. Natural polymers have gained prominence in this field, particularly in the formation of hydrogels, which serve as artificial scaffolds for TE. This section explores the role of herbal compound delivery in TE and the utilization of natural polymers for this purpose.

#### Hyaluronic acid

Because of its biocompatibility and special properties, hyaluronic acid has been increasingly applied in biomedical fields. Chemical modification of HA has lately become a useful carrier for TE and medicine distribution [315]. For instance, Luckanagul et al., (2021) delivered curcuma by using HA in a nanogel formulation. Although this work concentrated on drug delivery, tissue engineering potential of HA-grafted poly(N-isopropylacrylamide) still presents an interesting field for development [316]. The following models were used in haematological analyses: CUR-HA-pNIPAM 05 nanogel therapy, HA-pNIPAM 05 nanogel without drug, nanogel medium control, and treated skin model (negative control). The results are shown in Fig. 6B. It is clear from Fig. 6B that the handling procedure damaged the tissue integrity between the polycarbonate membrane and stratum basal in all samples. The stratum corneum, the outermost layer of skin, was also impacted [316].

#### Chitosan

Chitosan, derived from alkaline deacetylation of chitin, has demonstrated promise in tissue engineering applications. One of its notable benefits is its potential to reduce scarring, as investigated by Kumar Reddy et al. (2022). The utilization of chitosan-based scaffolds in TE can offer an ideal setting for tissue repair and regeneration [317].

#### Fibrin-

The scaffold structure plays a pivotal role in facilitating interactions between cells, tissues, and bioactive molecules in tissue engineering. Fibrin, a natural hydrogel-forming polymer, has been recognized for its ability to mimic essential parts of normal tissue and gather ECM parts [306] (Table 4). Scaffolds with high porosity and biodegradability, such as those based on fibrin, have demonstrated excellent performance in tissue engineering applications.

#### Nanoparticle-enhanced 3D printing

Combining medication-loaded NPs with 3D printing technology offers precise spatial and temporal drug release capabilities, making it an intriguing approach for tissue engineering. Monavari et al. (2021) utilized mesoporous silica-calcia nanoparticles (MSN) to transport an osteogenic medication, icariin, for a 3D printed osteogenic framework. The nanoparticles enhanced scaffold stiffness, promoted bioactivity, and efficiently delivered icariin, leading to improved osteoblast differentiation [309]. The image in Fig. 6C (a) displays the structure of the cells that have attached to the surface of the hydrogel structures. The DAPI and phalloidin stained photos suggest that the ADA-GEL constructs have a reduced cell density compared to the nanocomposite creations. This is likely because the unstable and weak ADA-GEL material is being destroyed more extensively. In contrast, SEM photos clearly illustrate that the cells have an elongated shape and cover a large area on the surface of the hydrogel structures [309]. This approach holds great potential for bone TE.

Jingjing Luo and colleagues introduced a novel approach to fabricating a structural/compositional gradient nano-/microfibrous mesh. They employed a co-electrospinning technique, combining silk fibroin-poly(ε-caprolactone) (SF-PCL) nanofibers with PCL microfibers. The researchers conducted additional analysis on the compressive modulus of the 3D scaffolds made of nano-/microfibers, comparing the scaffolds made of nanofibers to those made of microfibers. According to Fig. 6D, the nanofibrous cylinder scaffold had the highest compressive modulus of about 6.5 MPa. In comparison to the pure microfibrous scaffold, whose compressive modulus was 1.45 MPa, the nano-/microfibrous scaffold had a value of around 2.3 MPa. Therefore, the gradient nano-/microfibrous scaffold increased the compressive modulus compared to the pure rolled microfibrous scaffold [312].

This method allowed for the creation of a gradient mesh with varying structural and compositional characteristics, potentially offering unique properties for applications in tissue engineering, drug delivery, or other biomedical fields.

#### Silk fibroin

A natural biopolymer with exceptional characteristics, SF finds use in a wide range of industries. SF has been approved by the FDA for its compatibility with the human body, biodegradability, and electrical properties. Farahani et al. (2023) discuss the diverse applications of SF, including its use in tissue engineering for the heart, skin, cartilage, and medication transportation for tumor treatment [318]. SF's compatibility with other materials allows for tailored properties, such as improved hydrophilicity, electrical conductivity, and mechanical characteristics.

#### Hydrolysed Halomonas levan

Hydrolysed Halomonas levan (hHL), derived from levan polysaccharide produced by *Halomonas smyrnensis* halophilic bacteria, represents a novel biomaterial with potential applications in tissue engineering [313]. This hydrolysed derivative offers unique characteristics that could aid in TE, making it a candidate worth exploring in tissue engineering research.

### In vitro and in vivo results

The nano delivery of natural medication for tissue engineering has shown promising results across various formulations and compounds. The overview of reported literature is shown in Table 4. In the case of curcumin, diverse nano delivery methods have been employed. Verma et al. (2019) utilized CA nanocarriers, demonstrating anti-cancer, anti-inflammatory, and bone regenerative effects. Maroufi et al. (2021) introduced a novel CS/oxidized-modified quince seed gum/CUR-loaded HNTs hydrogel for tissue engineering [297]. ZSM-5 nanozeolite was combined into PCL/Gelatin nanofibers, showcasing sustained delivery efficiency (Table 4) [305]. In another approach, Narouiepour et al. (2022) employed niosomes for curcumin delivery, exhibiting enhanced recovery from traumatic brain injury in a rat model (Table 4) [298]. These studies involved in vitro assays, including cytotoxicity assessments on specific cell lines such as MG-63 human osteosarcoma cells, NIH3T3 fibroblast cells, and NS/PCs from the human fetal brain. The results consistently demonstrated the potential of nano delivery systems for curcumin in tissue engineering, combining anti-cancer, anti-inflammatory, and regenerative properties. In the case of icariin, various delivery systems and scaffolds have been explored. For the purpose of bone TE, Aghdam et al. (2021) designed an injectable CS hydrogel that incorporates altered HNTs. (Table 4) [308]. Additionally, icariin-loaded nano HAp supported with permeable framework were fabricated using alginate dialdehyde-gelatin with silica-calcia nanoparticles (Table 4) [309]. These formulations were evaluated using Human adipose-derived stem cells and human fetal osteoblasts, demonstrating their biocompatibility and potential for bone regeneration. Ginsenoside Rg1, delivered through SF-PCL nanofibers and PCL microfibers, exhibited positive effects in terms of morphology and compressive stress–strain test (Table 4) [312]. Resveratrol-loaded levan-based nanoparticles and levan hydrogels cross-linked with 1,4-Butanediol diglycidyl ether demonstrated biocompatibility and attachment promotion for human dermal fibroblast cell lines (PCS-201-012) and HaCaT cells, respectively (Table 4) [313, 314].

The overall results highlight the versatility and efficacy of nano delivery systems in tissue engineering applications. These systems offer targeted and sustained delivery of herbal compounds, showcasing their potential for diverse therapeutic outcomes in various tissue engineering contexts.

## Comparative analysis

Comparative study of many biomaterials for delivering herbal components reveals different advantages and disadvantages, each suitable for a different usage. Chitosan-based nanocarriers offer natural antibacterial properties and regulated release capacity even if they may demonstrate limited durability in acidic environments and lower loading capabilities. Hyaluronic acid-based nanocarriers have significant water solubility and targeted delivery potential, while their loading capacities for particular medicines are restricted and they may require additional targeting ligands. Liposomal nanoparticles struggle in large-scale manufacture and storage stability even if they show high biocompatibility and regulated release of both hydrophilic and hydrophobic substances. Although polymeric nanoparticles have gradual release and adjustable properties, their degradation products could induce inflammation or toxicity, and exhibit burst release kinetics. Although bacterial nanocellulose has restricted availability and more manufacturing costs, it presents excellent biocompatibility and mechanical strength together with prolonged release potential. Hydrogels—including chitosan and hyaluronic acid variants—offer high water content, tuneable properties, and sustained release potential even if mechanical strength and fast in vivo disintegration have possible constraints. Table 5 highlights this comparison, therefore directing the selection of appropriate biomaterials for the delivery of herbal chemicals depending on specific application needs. Although co-delivery of numerous herbal compounds provides opportunities for enhanced therapeutic advantages, it also requires careful formulation strategy and compound interaction studies.

**Table 5:** Comparative evaluation of different biomaterials for herbal compound delivery

Biomaterial	Advantages	Limitations	Representative herbal compound applications	Application area
Chitosan-based nanoparticles	Inherent antibacterial properties; biocompatible and biodegradable; controlled release	Limited stability in acidic environments; lower loading capacity	Allicin (garlic) in folate–PEG–chitosan NPs for enhanced antimicrobial activity; Curcumin for improved absorption and sustained anti-inflammatory effect	Antibacterial, anti-inflammatory
Hyaluronic acid-based nanoparticles	High water solubility and CD44-mediated targeting	Requires additional targeting ligands for specificity; low capacity for hydrophobic drugs	Quercetin for CD44 + tumor targeting; Ginsenoside Rg3 for tumor retention and enhanced apoptosis	Anticancer
Liposomal nanoparticles	Biocompatible; stable for both hydrophilic/lipophilic compounds; long circulation time	Scalability and storage limitations; moderate drug loading	Carvacrol and Thymol (oregano) for mucosal retention and enhanced antimicrobial action; Resveratrol for enhanced solubility and anti-tumor effect	Antibacterial, anticancer
Polymeric nanoparticles	Tunable release profiles; encapsulates diverse actives; customizable surfaces	Burst release risk; potential inflammatory degradation products	Baicalein (Scutellaria baicalensis) in ZIF-8–PDA for chemo–photothermal synergy; Berberine in PEG–PLA for improved bioavailability	Anticancer, antibacterial
Bacterial nanocellulose	High mechanical strength and biocompatibility; large surface area for loading	High cost; limited scalability for bulk production	Honey incorporated for moist wound healing; Aloe vera for epithelial regeneration and anti-inflammation	Wound healing
Hydrogels (Chitosan, HA, etc.)	High water content; tunable mechanics; biocompatible for tissue applications	Mechanical weakness; may degrade rapidly in vivo	Curcumin–HA hydrogel enhances VEGF/TGF-β for tissue repair; Centella asiatica–chitosan hydrogel promotes collagen and fibroblast activity	Wound healing, tissue regeneration

## Clinical trials

In the realm of clinical trials, it's imperative to understand the distinct regulatory framework imposed by the US Food and Drug Administration (FDA) on herbal remedies, pharmaceuticals, and supplements, categorizing them as food supplements. This categorization subjects them to regulatory criteria distinct from those governing conventional foods and drugs [319]. Particularly new herbal supplements avoid premarket approval and go against the thorough FDA medication review process [320]. Medications are manufactured using either "top-down" or "bottom-up" techniques, which permits modification for certain clinical uses [321]. As Guo in 2023 clarifies, despite developments, problems in optimizing delivery methods to solve issues including nanotoxicity and substance behaviour within living entities still exist [322]. Still, nanotechnology has great potential to boost the efficacy of herbal bioactive compounds in systems of medical therapy. For example, lately nanoemulsions have become champions of solubility, stability, and bioavailability of such drugs [323]. Presenting good uses in treating diseases like cancer, nanoparticle-based herbal drugs have showed promise in enhancing solubility, bioavailability, and therapeutic efficiency [321, 322, 324]. Furthermore under investigation are nanoparticles as means of delivering herbal treatments to enhance pharmacokinetics and cellular absorption [325]. Notable pharmacological nanotechnologies like phytosomes, nanoparticles, and self-emulsiating drug delivery systems have been recognized for their usefulness in delivering herbal bioactives, hence boosting their effectiveness [326]. In light of these findings, it is evident that nanotechnology holds substantial promise for enhancing the efficacy of herbal bioactive substances within medicine delivery systems. Table 6 shows an overview of some clinical trials of materials and herbal agents reviewed in this paper.

**Table 6:** anticancer and antibacterial clinical trials of herbal drugs

Name of the compound	Bioactive compound	Disease	Number of patients	Treatment	Results	Conclusion	Refs.
Garlic	Alicin	Gastric cancer	A study on 3365 high-risk individuals found that 2258 tested positive for H pylori and were arbitrarily allocated to treatment, vitamin, garlic, or placebos, while 1107 tested negative	Clinical trial	151 gastric cancer incidents and 94 deaths were identified between 1995–2017. Early effects of H. pylori treatment and vitamin supplementation on gastric cancer incidence and mortality appeared	H. pylori treatment and vitamin or garlic supplementation for over 22 years significantly reduced the risk of gastric cancer death	[327]
Garlic	Allicin	Infected mature anterior teeth	66 male patients were randomly divided into three groups (n = 22)1.Treated with Allium sativum2.Treated with calcium hydroxide3. Combination therapy	Clinical trial	Garlic exhibited a substantial decrease in Enterococcus faecalis FC in S3 and S4, in comparison to Ca (OH)2 and the combination of Ca (OH)2 + garlic. The combination shown the least notable reduction	The study found garlic intracanal medicine is effective in fighting Streptococcus bacteria, but more potent against Enterococcus faecalis species compared to Ca (OH)2	[328]
Honey	Manuka honey	Recalcitrant chronic rhinosinusitis	Twenty-five patients completed the study1. Treated with manuka honey (n = 10)2.Control group (n = 15)	Clinical trial	The study found that 60% of patients treated with manuka honey showed a decrease in bacterial culture rates, while 80% of the control group showed no significant change	The study suggests that twice-daily 16.5% MH supplemented with 1.3 mg/mL MGO sinonasal rinses is safe, but not as efficacious as culture-directed oral antibiotics and twice-daily saline rinses	[329]
Resveratrol	Resveratrol	Reducing the proportion of patients experiencing grade ≥ 3 toxicity from 90 to 70% in advanced gastric cancer	A total of 30 patients, with a median age of 54 years, were included in the study, of which 73% were male	Phase II single-arm clinical trial	Resveratrol-copper (R-Cu) treatment did not lower the overall cumulative incidence. of grade ≥ 3 toxicity (77%), or of ≥ 3 haematological toxicity (73%)	The study found that patients receiving R-Cu showed a significant reduction in non-haematological toxicities, but this did not impact the progression-free survival or overall survival outcomes	[330]
Curcumin	Curcumin	indirect pulp capping in permanent molar teeth	A study on 100 decayed human permanent molars categorized them into five groups: IPC, CHX gel, NaOCl, methylene blue-mediated antimicrobial photodynamic treatment, and curcumin-mediated antimicrobial PDT	Clinical trial	CUR-mediated antimicrobial PDT treated samples demonstrated the highest four-point bending strength scores (70 ± 18 MPa)	The utilization of antimicrobial PDT, particularly with MB mediation, exhibited enhanced shear bond strength, micro-tensile bond strength, and four-point bending strength measurements	[331]
Turmeric	Curcumin	Breast Cancer	Patients (n = 26) consumed 3 capsules/day (turmeric, red clover, and flaxseed extracts plus resveratrol; 296.4 mg phenolics/capsule) from biopsy-confirmed diagnosis to surgery (5 ± 2 days) or did not consume capsules (n = 13)	Clinical trial	Curcuminoids in breast tissue inhibit p53-wild-type MCF-7 cell growth through the p53/p21 pathway, while isoflavone-generated metabolites exhibit estrogen-like effects	Regular ingestion of curcuminoids may serve as coadjuvants in the battle against breast cancer	[332]
Genistein	Genistein	prostate cancer	American men with localized prostate cancer (PCa) underwent treatment with genistein (N = 14) compared to those not receiving treatment (N = 14) for one month before radical prostatectomy	phase II trial	Genistein treatment initially reduced MMP-2 levels in PC3 human prostate cancer cells, but prolonged treatment increased MMP-2 levels while maintaining cells' anti-motility response	Genistein inhibits prostate-specific pathways causing lethal high motility, leading to compensatory changes in efficacy biomarkers over long-term treatment	[333]
Honey	Manuka honey-impregnated dressings	Neuropathic diabetic foot ulcers (NDFU)	A total of 63Caucasians, type 2 diabetic patients were participatedgroup I patients were treated with MHID and group II patients were treated withconventional dressings (CD)	Clinical trial	In group I, the average time it took for ulcers to cure was 31 ± 4 days. Additionally, during the first week, 78·13% of ulcers in group I patients were sterile. In the following weeks (2, 4, and 6), the proportion of ulcers becoming sterile varied	MHID is an efficient treatment for NDFU, resulting in a substantial decrease in healing time and quick cleaning of ulcers	[334]
Genistein	Genistein	Metastatic colorectal cancer	Thirteen patients received chemotherapy with Genistein in this trial. Subjects were treated with FOLFOX or FOLFOX-Bevacizumab. Genistein was administered orally for 7 days every 2 weeks	phase I/II pilot study	Genistein alone caused minor side effects, but when combined with chemotherapy, no negative effects occurred. The highest response rate and median progression-free survival were 61.5% and 11.5 months, respectively	The addition of Genistein to FOLFOX or FOLFOX-Bevacizumab was found to be safe and well tolerated	[335]
Scutellaria baicalensis	Baicalin	Acute lymphocytic leukemia	26 patients with Acute lymphocytic leukemia were treated and compared with a healthy control group	Clinical trial	Scutellaria extract (SBE) enhances immune system function by increasing IFNγ production in PBLs, reducing TNFα and IL-10 levels in BMCs, and exhibiting pro-apoptotic properties	Scutellaria extract effectively reduced PBL viability in acute lymphoblastic leukemia patients, controlled cytokine generation, enhanced resistance, and triggered apoptosis, without affecting healthy control leukocytes	[336]
Hyaluronic acid	Hyaluronic acid	Knee osteoarthritis	122 knees were randomly distributed into HA (34 knees), PRP (40 knees), and PRP + HA (48 knees) groups	Clinical trial	At the 24-month mark, the PRP + HA group exhibited enhanced pain and function scores, while experiencing improved synovial hyperplasia at 6 and 12 months. On the other hand, the PRP group saw the most significant problems	The combination of PRP and HA is superior to PRP or HA alone in suppressing synovial inflammation. Moreover, this combination significantly enhances pain relief, improves functionality, and minimizes adverse responses	[337]
Quercetin	Quercetin	Prostate cancer	Thirty-one men with prostate cancer consumed daily 1 g of green tea extract (GTE) (830 mg of green tea polyphenol (GTP)) with 800 mg of quercetin (Q) (GT + Q) or placebo (GT + PL) for four weeks before prostatectomy	Prospective, randomized, parallel design, placebo controlled trial	The group receiving GT + Q exhibited a reduction in urinary and plasma EGC levels two hours after taking the capsule, in comparison to the GT + PL-group	The administration of 800 mg of Q supplementation and 1000 mg of GTE before prostatectomy did not significantly increase EGCG and ECG concentrations or decrease methylated GTPs in prostate tissue	[338]
Aloe vera	Aloe vera	Traumatic oral ulceration (TOU)	A study was undertaken in a university context, comprising 140 patients aged 12 and above, to evaluate the effectiveness of Aloe Vera or CHX gel in fixed orthodontic therapy	double‑blind randomized clinical trial	43.6% of patients had TOUs, with Aloe Vera gel yielding superior outcomes compared to CHX. Aloe Vera had a reduced risk and treatment count in comparison to CHX, with a notable disparity in the occurrence of tooth and oral ulcerations (TOUs)	The results suggest that the administration of Aloe Vera gel to patients with fixed orthodontic appliances could play a vital role in effectively preventing TOU	[339]
Calendula	Calendula officinalis L	Acute wounds	Two cohorts of 20 patients with hand and finger skin loss were treated using the secondary intention method, with the control group receiving mineral oil and the intervention group receiving SEC	Controlled randomized clinical trial	The time it took for epithelization and the pace of healing were quicker in the IG group compared to the CG group (IG = 8.6 ± 4.7 days and 9.5 ± 5.8% day, ƿ < 0.05)	Healing by secondary intention in acute hand and finger wounds with SEC led to expedited epithelialization	[340]
Epigallocatechin-3-Gallate	Epigallocatechin-3-Gallate	Preventing Dermatitis in Patients With Breast Cancer	A total of 180 eligible patients were enrolled, of whom 165 (EGCG, n = 111; placebo, n = 54) were evaluable for efficacy (median [range] age, 46 [26–67] years)	Double-Blind, Placebo-Controlled, Phase 2 Randomized Clinical Trial	The EGCG group showed significantly lower grade 2 or worse RID, mean RIDI, and symptom indexes compared to the placebo group	EGCG solution significantly reduced radiation-induced dermatitis in breast cancer patients receiving adjuvant radiotherapy, indicating its potential as a novel skincare option	[341]

## Challenges and future perspectives

Herbal compound delivered via biomaterials provide numerous difficulties that demand consideration for next developments in the sector. A main difficulty is reaching ideal distribution efficiency in view of stability, bioavailability, and targeted delivery to certain places. Biomaterial-based delivery systems have to solve problems including restricted loading capacity, burst release kinetics, and possible toxicity related with breakdown products. Constant research on creative approaches including surface modification of biomaterials, inclusion of targeted ligands, and development of stimuli-responsive delivery systems helps one to overcome these obstacles. Furthermore, developments in nanotechnology present interesting paths to improve the effectiveness of herbal compound distribution by means of nanocarriers with exact control over size, form, and surface qualities [342].

Nanocarriers have shown great potential for regenerative medicine and cancer therapy by influencing, modulating, and controlling the immune system on one hand, and playing an effective role in gene delivery on the other hand [343–345].

Looking ahead, the field of herbal ingredient administration is primed for major expansion with developing themes probably including personalized medicine methods, combination medicines, and integration with modern imaging and diagnostic technologies. Although nanotechnology has shown great potential to enhance the bioavailability of natural compounds, clinical trials remain limited. Several challenges need to be addressed in both preclinical and clinical stages, including large-scale production, ensuring long-term stability of nanotherapeutics, overcoming physiological barriers, and resolving safety and regulatory concerns [346].

By the way, combining conventional chemotherapeutic agents with natural compound-based drugs in nanocarrier delivery systems may produce synergistic effects in cancer therapy.[347, 348]. Moreover, the concurrent administration of nanoparticles loaded with herbal drugs alongside those containing conventional antibiotics represents a promising strategy for antibacterial therapy and wound healing, particularly in cancer patients [349, 350]. Furthermore, applying physical stimuli, such as electric fields, may enhance the efficacy of nanocarriers delivering herbal drugs and natural products in antibacterial and anticancer applications [351–355]. The field has great potential to transform healthcare by means of the efficient use of herbal components for different medicinal purposes by solving present problems and seizing future prospects.

## Conclusion

Drawing on observations of nature, herbal remedies have been the cornerstone of healing. But because to technological developments, manufactured drugs—which had improved efficacy but carried side effects—became rather common. A return to traditional herbal therapy was taken as synthetic drugs neared a hazardous level. Covering their several applications in antibacterial, anticancer, wound healing, and regenerative medicine domains, this review has investigated the intricate network of herbal compounds. However, conventional administration methods limit the ability of herbal medication to carry active components and possible adverse effects, therefore reducing its efficacy. Targeted therapy and support to arrest deterioration are promised by nanoparticles, liposomes, and nanoemulsions among nanodelivery techniques. In medicine, stressing accuracy and customized treatment; this mix of conventional herbal knowledge with nanotechnology is transforming treatment. Advancement depends on multidisciplinary cooperation since it provides means to produce tailored treatments and improve patient outcomes. Emerging as change agents in herbal medicine, biomaterials are advancing general safety and efficacy by themselves. Our responsibility is to protect and forward this information so that it may always be readily available and continuing growing globally. In conclusion, this review highlights the importance of nanocarrier systems for developing the clinical utility of active herbal constituents with demonstrated therapeutic efficacy. Through a selective set of successful examples, we illustrated how nano-delivery platforms improve pharmacokinetics, cellular uptake, and disease-specific efficacy of phytochemical agents in anticancer, antimicrobial, and regenerative contexts. The formulation processes, experimental models, and outcomes are detailed in Tables 1 and 2, as relating to the translational applicability of nanoformulated herbal agents. By using only the most validated examples of herbal-nanocarrier strategies, we intended to illustrate the practical way forward for traditional phytomedicine to be integrated with contemporary nanotechnology-based therapeutics.

## Data Availability

Not applicable.
